# Systematic review and network meta-analysis comparing palbociclib with chemotherapy agents for the treatment of postmenopausal women with HR-positive and HER2-negative advanced/metastatic breast cancer

**DOI:** 10.1007/s10549-017-4404-4

**Published:** 2017-07-27

**Authors:** Florence R. Wilson, Abhishek Varu, Debanjali Mitra, Chris Cameron, Shrividya Iyer

**Affiliations:** 1 Cornerstone Research Group Inc., Suite 204, 3228 South Service Road, Burlington, ON L7N 3H8 Canada; 2 Pfizer, Inc., New York, NY USA

**Keywords:** Advanced/metastatic breast cancer, Chemotherapy, Network meta-analysis, Palbociclib, Progression-free survival, Time to progression

## Abstract

**Purpose:**

To compare palbociclib + letrozole and palbociclib + fulvestrant with chemotherapy agents in postmenopausal women with hormone receptor-positive (HR+)/human epidermal growth factor receptor 2-negative (HER2−) advanced/metastatic breast cancer (ABC/MBC) who had no prior systemic treatment for advanced disease (first line) or whose disease progressed after prior endocrine therapy or chemotherapy (second line).

**Methods:**

A systematic search identified randomized controlled trials (RCTs) published from January 2000 to January 2016 that compared endocrine-based therapies, chemotherapy agents, and/or chemotherapy agents + biological therapies in the first- and second-line treatment of postmenopausal women with HR+/HER2− ABC/MBC. The main outcome of interest was progression-free survival (PFS)/time to progression (TTP). Bayesian network meta-analyses (NMAs) and pairwise meta-analyses were conducted. Heterogeneity and inconsistency were assessed.

**Results:**

Sixty RCTs met eligibility criteria and were stratified by line of therapy. In the first line, palbociclib + letrozole showed statistically significant improvements in PFS/TTP versus capecitabine [intermittent: HR 0.28 (95% CrI 0.11–0.72)] and mitoxantrone [HR 0.28 (0.13–0.61)], and trended toward improvements versus paclitaxel [HR 0.59 (0.19–1.96)], docetaxel [HR 0.51 (0.14–2.03)] and other monotherapy or combination agents (HRs ranging from 0.24 to 0.99). In the second line, palbociclib + fulvestrant showed statistically significant improvements in PFS/TTP versus capecitabine [intermittent: HR 0.28 (0.13–0.65)], mitoxantrone [HR 0.26 (0.12–0.53)], and pegylated liposomal doxorubicin [HR 0.19 (0.07–0.50)], and trended toward improvements versus paclitaxel [HR 0.48 (0.16–1.44)], docetaxel [HR 0.71 (0.24–2.13)] and other monotherapy or combination agents (HRs ranging from 0.23–0.89). NMA findings aligned with direct evidence and were robust to sensitivity analyses.

**Conclusions:**

Palbociclib + letrozole and palbociclib + fulvestrant demonstrate trends in incremental efficacy compared with chemotherapy agents for the first- and second-line treatment of HR +/HER2− ABC/MBC.

**Electronic supplementary material:**

The online version of this article (doi:10.1007/s10549-017-4404-4) contains supplementary material, which is available to authorized users.

## Introduction

Postmenopausal women with hormone receptor-positive (HR+), human epidermal growth factor receptor type 2-negative (HER2−) tumors represent the majority of patients with advanced/metastatic breast cancer (ABC/MBC) [1–3]. Despite the sometimes indolent course of the disease, HR+/HER2− ABC/MBC remains incurable [1–3]. Guidelines suggest that endocrine therapy should be offered as standard first-line treatment in patients who do not have visceral crises [1–3]. After receiving first-line endocrine therapy, many patients experience disease progression due to endocrine resistance and are offered chemotherapy as second-line therapy [2]. Various monotherapy and combination chemotherapy regimens are available, providing treatment options for patients with endocrine resistance [4].

Palbociclib (IBRANCE^®^; Pfizer Inc, New York, NY, USA) is a new oral cyclin-dependent kinase 4/6 (CDK4/6) inhibitor approved by the United States (US) Food and Drug Administration (FDA) for HR+/HER2− ABC/MBC in combination with letrozole as initial endocrine-based therapy [5], or in combination with fulvestrant for patients whose disease had progressed following prior endocrine therapy [6]. The efficacy and safety of palbociclib combination therapies have been demonstrated in phase 3 clinical studies [5, 6]; however, a comparison of progression-free survival (PFS) has not been made between palbociclib and chemotherapy agents. Here, we report the results of a systematic literature review (SLR) and network meta-analysis (NMA) that evaluates the efficacy of palbociclib + letrozole and palbociclib + fulvestrant versus chemotherapy agents in postmenopausal women with HR+/HER2− ABC/MBC who had no prior systemic treatment for advanced disease (first line) or whose disease had progressed after prior endocrine therapy or chemotherapy (second line).

## Methods

### Systematic literature review

An SLR was conducted to identify randomized controlled trials (RCTs) published from January 2000 to January 2016. All references used in two previous NMAs by Generali et al. [7] and Chirila et al. [8] formed the starting point for the current systematic review. These represent the most recent NMAs conducted for chemotherapy agents and for endocrine therapies. The NMA by Generali et al. [7] compared everolimus + exemestane with various chemotherapy agents, and the literature search spanned from 2000 to May 2014. The NMA by Chirila et al. [8] compared palbociclib with other endocrine-based therapies, and the literature search was conducted in January 2015 with no date restrictions. An updated literature search was performed by searching MEDLINE, EMBASE, Cochrane CENTRAL, and PubMed from May 2014 (search date of Generali) to January 2016 to identify RCTs that were published since the aforementioned two reviews. A predefined search strategy (Online Appendix A) was used, based on the previous searches by Generali et al. [7] and Chirila et al. [8]. The search was designed to identify all RCTs of chemotherapy agents, chemotherapy agents + biological therapies, and endocrine therapies used to treat postmenopausal women with HR+/HER2− ABC/MBC who had not received any prior systemic anticancer treatment for advanced disease (first line) or whose disease had progressed after prior endocrine therapy or chemotherapy (second line). However, the current analysis focuses only on chemotherapy agents.

Predefined eligibility criteria were used to screen all identified studies (Online Appendix B; additional details are available upon request). Phase 2 and phase 3 RCTs and conference abstracts were included. Treatments of interest included chemotherapy agents, chemotherapy agents + biological therapies, and endocrine-based therapies. Endocrine-based therapies were included in all analyses but have not been reported here, given that the focus of this analysis is on chemotherapy agents. Outcomes of interest were PFS, time to progression (TTP), and overall survival (OS), reported as hazard ratios (HRs) with 95% confidence intervals (CIs). PFS and TTP were considered as equivalent outcomes since the definitions aligned well across studies and any heterogeneity was considered non-substantial. As the outcome of disease progression is a negative event for patients, HRs < 1 corresponded to beneficial treatment effects of the first treatment compared with the second treatment. The analysis of OS has been excluded here due to lack of availability of final OS data from the palbociclib clinical trials.

Two reviewers independently reviewed citation titles and abstracts identified in the updated literature search to assess study eligibility. Citations considered to describe potentially eligible articles were independently reviewed in full-text form. A PRISMA flow diagram documenting the process of study selection was prepared.

### Network meta-analysis

Network meta-analysis is a widely used approach to derive estimates of effect among treatments that may not have been compared directly in clinical trials. Bayesian NMAs and pairwise meta-analyses were conducted to pool RCT results using well-established methods outlined by the National Institute for Health and Care Excellence (NICE) [9, 10]. Two separate evidence networks were generated to stratify studies by first and second lines of therapy. Based on the line of therapy definitions used in the palbociclib clinical trials [5, 6], first line of therapy was defined as having neither previous systemic endocrine therapy nor chemotherapy for ABC/MBC, and second line of therapy was defined as having previous systemic endocrine therapy or chemotherapy for ABC/MBC.

For each pairwise comparison, HRs with 95% credible intervals (CrIs) were used as a measure of the association between the treatment and its efficacy. Estimates with 95% CrIs that excluded the null value of 1 were considered to reflect statistically significant differences between interventions. Additional measures of effect were also generated, including Surface Under the Cumulative RAnking curve (SUCRA) values (expressed as percentages, which show the relative probability of an intervention being among the best options), probability best, and mean rank [11]. For interpretation, SUCRA values and probability best range between 0 and 1, with values closer to 1 being preferred [11].

Fixed-effects models were performed as primary analyses, given that the networks are largely composed of single-study connections. Random-effects models were performed as secondary analyses, using both informative and vague priors on the variance. Informative priors were based on an estimate of between-study variance using data from previous Cochrane systematic reviews [12]. For vague priors, we assumed a uniform distribution [i.e., Uniform (0, 5)] for between-study variance, as recommended by the NICE [9]. To assess whether the models had adequate fit to the data, the posterior residual deviance from each NMA was compared to the corresponding number of unconstrained data points; approximately equal values represented an adequate fit.

Network meta-analyses were performed using WinBUGS (version 1.4.3) and R (version 3.2.2) and were based on burn-in samples of at least 40,000 iterations and subsequent sampling iterations of at least 50,000 iterations (WinBUGS code is available upon request). Trace plots and Gelman–Rubin plots were reviewed to assess model convergence.

### Assessment of heterogeneity and inconsistency

In accordance with the exchangeability assumption of NMAs [13], study and patient characteristics were assessed to ensure similarity and to investigate the potential impact of heterogeneity on effect estimates. Factors considered included mean/median age, HR status, HER2 status, menopausal status, prior therapies, crossover after disease progression, blinding, drug dosing, and endpoint definitions. Heterogeneity was assessed by summarizing relevant information using tables and by conducting sensitivity analyses where possible. The presence of several single-study connections between interventions in the evidence networks precluded us from performing meta-regression analyses or sub-group/sensitivity analyses related to certain characteristics of interest [14]. Sensitivity analyses were conducted to include both the palbociclib phase 2 and 3 studies [5, 15], and to adjust for heterogeneity in median PFS/TTP values.

The NMA results were qualitatively compared with pairwise estimates generated from traditional frequentist meta-analyses of direct evidence. Inconsistency in the networks was assessed by comparing deviance and deviance information criterion (DIC) statistics in fitted consistency and inconsistency models [16]. The posterior mean deviance of the individual data points in the inconsistency model was plotted against the corresponding posterior mean deviance in the consistency model to identify any loops where inconsistency was present (available upon request).

## Results

### Study selection

The NMA by Generali et al. [7] included 44 RCTs, which were not stratified by line of therapy. The NMA by Chirila et al. [8] included 27 RCTs, stratified by line of therapy. Of these, 53 RCTs met the eligibility criteria described above. In addition, two recently published studies that provided updated PFS results for palbociclib trials in first line [5] and second line [6] were included. Among the 2600 study records that were identified in the updated literature search, seven RCTs met the eligibility criteria and were included in the NMA.

**Fig. 1:**
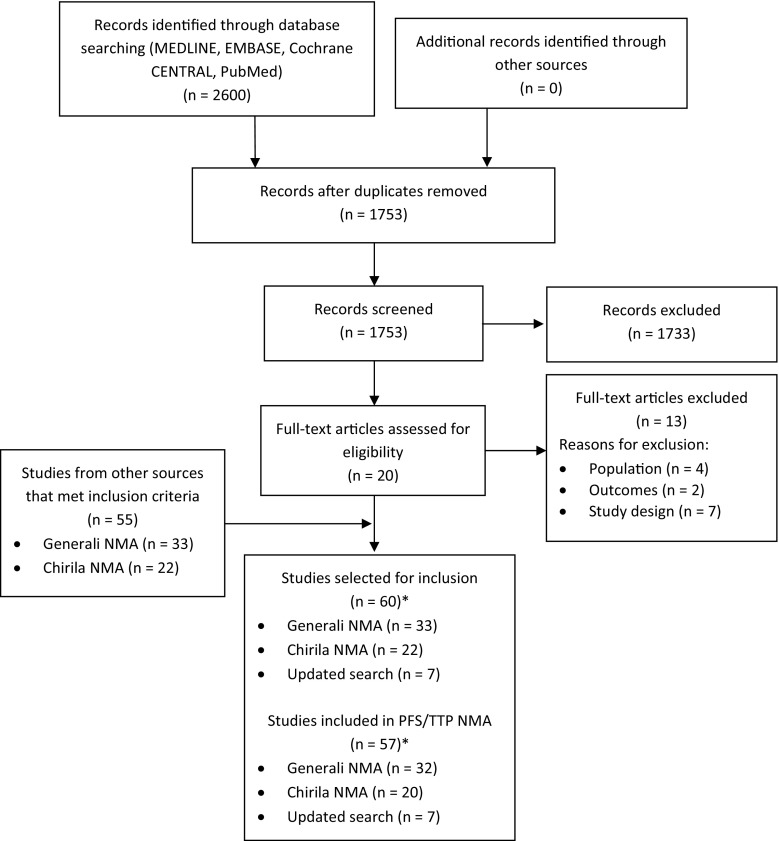
PRISMA flow diagram *NMA* network meta-analysis, *PRISMA* preferred reporting items for systematic reviews and meta-analyses. *Two studies overlapped between the Generali et al. NMA and the Chirila et al. NMA

In total, 60 RCTs (from the three SLRs) met the eligibility criteria; however, only 57 RCTs were included in the PFS/TTP NMA that is presented here (Fig. 1). In order for the evidence networks to be fully connected, some connections had to be forced based on line of therapy and patient characteristics. The three connections forced based on patient characteristics were chemotherapy trials and were due to less than 50% of patients being HR+ [17–19].

### Study and patient characteristics

The 57 RCTs included in analyses were published between 1992 and 2016, with trials conducted on all continents. Mean age across the trials ranged from 51 to 70 years, and median follow-up ranged from 6 to 61.2 months (Online Appendix C). The percentage of HR+ patients was reported in 56 of the 57 trials and ranged from about 15 to 100%, and the proportion of patients receiving prior metastatic endocrine therapy or chemotherapy ranged from 0 to 100%. Based on this high level of heterogeneity, trials were stratified by line of therapy based on prior neoadjuvant/adjuvant and advanced/metastatic therapy received by patients (details available upon request). Assessment of other study and patient characteristics revealed that many sensitivity analyses were not feasible due to insufficient information or disconnected evidence networks. Overall, the studies included in the NMA had a low risk of bias (Online Appendix D). A summary of the median PFS/TTP values and HRs used in analyses is available upon request.

### First-line therapy progression-free survival/time to progression

**Fig. 2:**
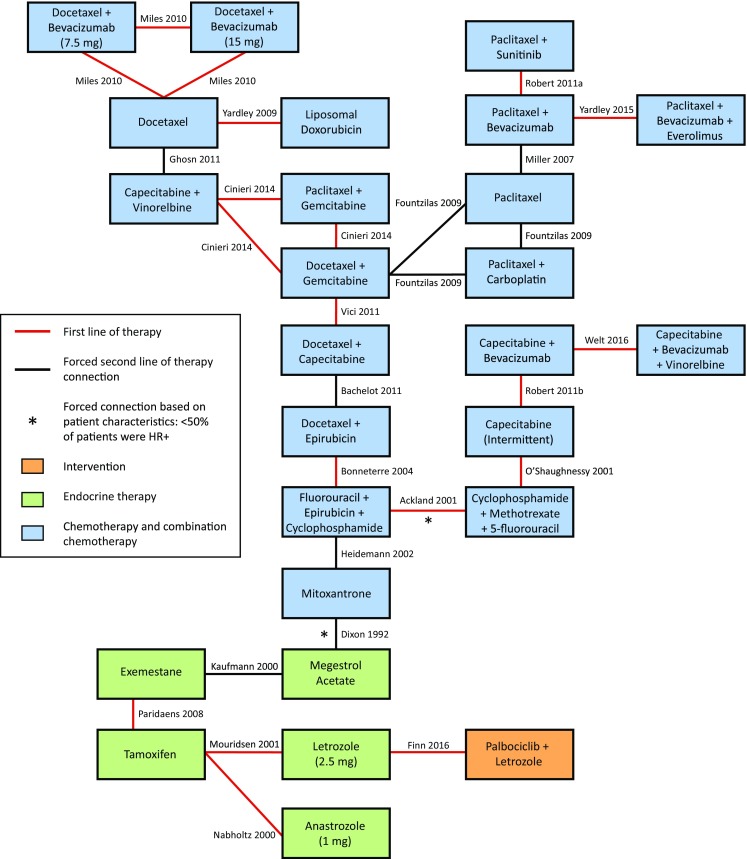
Evidence network for first-line PFS/TTP *HR*+ hormone receptor positive, *PFS* progression-free survival, *TTP *time to progression

The evidence network for the first-line PFS/TTP NMA is shown in Fig. 2. Each intervention is represented by a node and randomized comparisons are shown as links between the nodes. Overall, 22 studies were included that enrolled a total of 8152 patients with available outcomes data. Data from head-to-head trials were available for 28 pairwise comparisons in the network, with single studies informing all of these comparisons. This analysis includes data from the PALOMA-2 trial which compares palbociclib + letrozole with letrozole [5].

In the fixed-effects model, palbociclib + letrozole showed statistically significant improvements in PFS/TTP relative to capecitabine [intermittent: HR 0.28 (95% CrI 0.11–0.72)] and mitoxantrone [HR 0.28 (0.13–0.61)], and trended toward improvements (not statistically significant) versus paclitaxel [HR 0.59 (0.19–1.96)], docetaxel (HR 0.51 (0.14–2.03)], and other monotherapy or combination chemotherapy agents (HRs ranging from 0.24 to 0.99; Table 1). Palbociclib + letrozole ranked more favorably than all chemotherapy comparators for PFS/TTP in terms of SUCRA, probability best, and mean rank. Palbociclib + letrozole was associated with the highest SUCRA value among all treatments (96.00%), the highest probability of being the best treatment (41.70%), and a treatment ranking closest to 1. Model fit statistics from the fixed-effects model were favorable; a total residual deviance value close to the number of unconstrained data points was obtained (i.e., 25.08 vs. 25).

**Table 1:** First-line therapy NMA results for PFS/TTP: palbociclib + letrozole versus comparators

Comparisons	HR (95% CrI) Fixed-effects model	HR (95% CrI) Random-effects model: vague priors	HR (95% CrI) Random-effects model: informative priors
Palbociclib + letrozole	1	1	1
Single chemotherapy agents			
Paclitaxel	0.59 (0.19–1.96)	0.59 (0.07–4.83)	0.63 (0.07–5.48)
Docetaxel	0.51 (0.14–2.03)	0.50 (0.06–3.92)	0.55 (0.07–4.24)
Capecitabine (intermittent)	**0.28 (0.11**–**0.72)**	0.27 (0.03–2.12)	0.29 (0.03–2.45)
Mitoxantrone	**0.28 (0.13**–**0.61)**	0.27 (0.03–2.23)	0.28 (0.03–2.28)
Combination chemotherapy agents			
Paclitaxel + bevacizumab + everolimus	0.99 (0.29–3.81)	0.98 (0.12–8.43)	0.93 (0.12–7.17)
Paclitaxel + bevacizumab	0.98 (0.31–3.38)	0.96 (0.13–7.29)	0.94 (0.10–8.65)
Docetaxel + bevacizumab 15 mg	0.65 (0.18–2.69)	0.65 (0.09–4.70)	0.72 (0.09–6.11)
Docetaxel + bevacizumab 7.5 mg	0.59 (0.16–2.40)	0.58 (0.08–4.19)	0.64 (0.08–5.36)
Paclitaxel + sunitinib	0.60 (0.18–2.14)	0.60 (0.07–4.93)	0.66 (0.09–5.00)
Docetaxel + gemcitabine	0.59 (0.20–1.91)	0.59 (0.08–4.32)	0.64 (0.08–5.20)
Liposomal doxorubicin	0.54 (0.14–2.29)	0.53 (0.07–3.79)	0.59 (0.07–4.99)
Paclitaxel + gemcitabine	0.51 (0.15–1.87)	0.49 (0.06–4.21)	0.56 (0.08–4.06)
Paclitaxel + carboplatin	0.53 (0.17–1.83)	0.51 (0.06–4.35)	0.57 (0.06–5.16)
Docetaxel + capecitabine	0.51 (0.19–1.49)	0.50 (0.06–4.19)	0.54 (0.08–3.90)
Capecitabine + vinorelbine	0.50 (0.16–1.72)	0.49 (0.06–4.18)	0.55 (0.06–4.98)
Capecitabine + bevacizumab + vinorelbine	0.48 (0.19–1.27)	0.46 (0.06–3.76)	0.50 (0.06–4.02)
Docetaxel + epirubicin	0.47 (0.20–1.19)	0.46 (0.06–3.32)	0.49 (0.06–4.15)
Capecitabine + bevacizumab	0.40 (0.16–1.06)	0.39 (0.05–2.82)	0.41 (0.06–2.99)
Fluorouracil + epirubicin + cyclophosphamide	**0.33 (0.15**–**0.77)**	0.32 (0.04–2.47)	0.34 (0.03–3.34)
Cyclophosphamide + methotrexate + 5-fluorouracil	**0.24 (0.11**–**0.57)**	0.24 (0.03–2.11)	0.25 (0.03–1.85)
Model fit statistics	Residual deviance = 25.08 vs. 25 DIC = −0.02	Residual deviance = 25.71 vs. 25 DIC = 0.11 Heterogeneity (SD) = 0.67 (0.01–4.53)	Residual deviance = 25.69 vs. 25 DIC = −0.05 Heterogeneity (SD) = 0.73 (0.02–3.64)

Analyses use data from PALOMA-2 [5]. Statistically significant differences are shown in ***bold***. Endocrine therapies have been excluded from this table, given that the focus is on chemotherapy agents. For vague priors in the random-effects model, a uniform distribution for between-study variance was assumed, as recommended by the National Institute for Health and Care Excellence [9]. Informative priors were based on an estimate of between-study variance using data from previous Cochrane systematic reviews [12]

*Crl *credible interval, *DIC* deviance information criterion, *HR* hazard ratio, *SD* standard deviation

In the random-effects models using both informative and vague priors on the variance, palbociclib + letrozole trended toward improvements (not statistically significant) versus all chemotherapy comparators. Model fit was favorable and relatively constant across both analyses (Table 1).

### Second-line therapy progression-free survival/time to progression

**Fig. 3:**
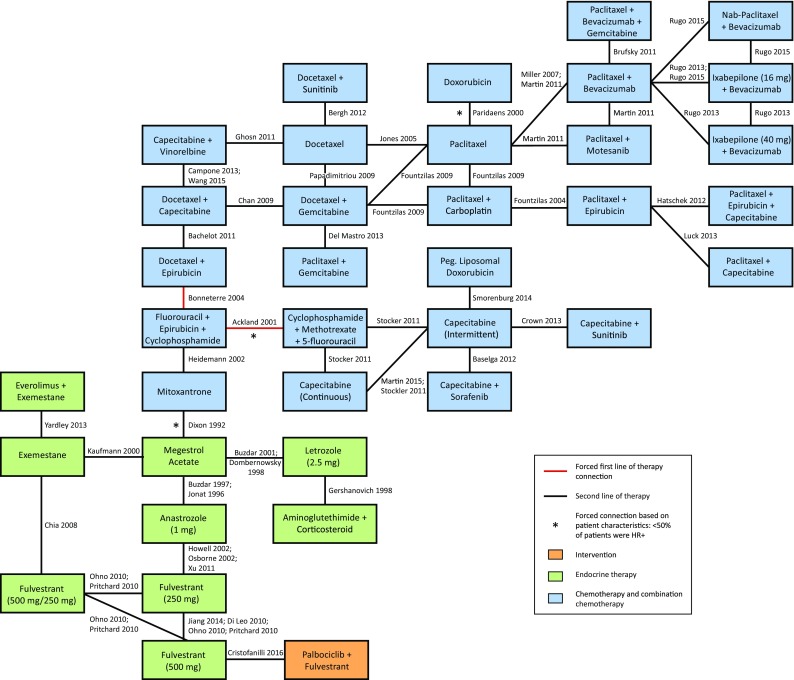
Evidence network for second-line PFS/TTP *HR*+ hormone receptor positive, *PFS *progression-free survival, *TTP* time to progression

Figure 3 presents the evidence network for the second-line PFS/TTP NMA. Overall, 44 studies were included that enrolled a total of 14,708 patients with available outcomes data. Data from head-to-head trials were available for 45 of the pairwise comparisons in the network, with single studies informing 35 of these comparisons. This analysis includes data from the PALOMA-3 trial which compares palbociclib + fulvestrant with fulvestrant 500 mg [6].

In the fixed-effects model, palbociclib + fulvestrant showed statistically significant improvements in PFS/TTP relative to capecitabine [intermittent: HR 0.28 (95% CrI 0.13–0.65); continuous: HR 0.24 (0.11–0.56)], mitoxantrone [HR 0.26 (0.12–0.53)], and pegylated liposomal doxorubicin [HR 0.19 (0.07–0.50)], and trended toward improvements (not statistically significant) versus paclitaxel [HR 0.48 (0.16–1.44)], docetaxel [HR 0.71 (0.24–2.13)], and other monotherapy or combination chemotherapy agents (HRs ranging from 0.23 to 0.89; Table 2). Palbociclib + fulvestrant ranked more favorably than all chemotherapy comparators for PFS/TTP in terms of SUCRA, probability best, and mean rank. Palbociclib + fulvestrant was associated with the highest SUCRA value among all treatments (97.20%), and an 18.90% probability of being the best treatment.

**Table 2:** Second-line therapy NMA results for PFS/TTP: palbociclib + fulvestrant versus comparators

Comparisons	HR (95% CrI) Fixed-effects model	HR (95% CrI) Random-effects model: vague priors	HR (95% CrI) Random-effects model: informative priors
Palbociclib + Fulvestrant	1	1	1
Single chemotherapy agents			
Doxorubicin	0.80 (0.27–2.44)	0.81 (0.22–3.02)	0.82 (0.19–3.40)
Docetaxel	0.71 (0.24–2.13)	0.69 (0.20–2.57)	0.70 (0.17–2.76)
Paclitaxel	0.48 (0.16–1.44)	0.49 (0.14–1.74)	0.49 (0.12–1.98)
Capecitabine (intermittent)	**0.28 (0.13–0.65)**	**0.29 (0.10–0.81)**	**0.29 (0.10–0.89)**
Mitoxantrone	**0.26 (0.12–0.53)**	**0.26 (0.11–0.60)**	**0.26 (0.11–0.65)**
Capecitabine (continuous)	**0.24 (0.11–0.56)**	**0.25 (0.09–0.70)**	**0.25 (0.09–0.78)**
Pegylated liposomal doxorubicin	**0.19 (0.07–0.50)**	**0.20 (0.06–0.64)**	**0.19 (0.06–0.67)**
Combination chemotherapy agents			
Paclitaxel + bevacizumab + gemcitabine	0.89 (0.28–2.82)	0.89 (0.23–3.49)	0.91 (0.20–3.95)
Docetaxel + sunitinib	0.77 (0.26–2.36)	0.75 (0.21–2.81)	0.76 (0.18–3.12)
Paclitaxel + bevacizumab	0.72 (0.24–2.20)	0.73 (0.20–2.68)	0.74 (0.17–3.11)
Paclitaxel + gemcitabine	0.64 (0.22–1.94)	0.67 (0.19–2.45)	0.67 (0.16–2.77)
Ixabepilone 40 mg + bevacizumab	0.61 (0.18–2.05)	0.62 (0.15–2.53)	0.62 (0.13–2.84)
Nab-paclitaxel + bevacizumab	0.60 (0.20–1.87)	0.60 (0.16–2.27)	0.61 (0.13–2.61)
Docetaxel + gemcitabine	0.55 (0.19–1.61)	0.57 (0.17–1.99)	0.58 (0.15–2.29)
Paclitaxel + motesanib	0.51 (0.16–1.61)	0.53 (0.14–2.13)	0.53 (0.12–2.33)
Docetaxel + capecitabine	0.49 (0.17–1.38)	0.51 (0.16–1.70)	0.51 (0.14–1.96)
Docetaxel + epirubicin	0.45 (0.19–1.06)	0.46 (0.17–1.28)	0.47 (0.16–1.44)
Paclitaxel + carboplatin	0.44 (0.14–1.38)	0.46 (0.13–1.67)	0.46 (0.11–1.94)
Ixabepilone 16 mg + bevacizumab	0.45 (0.15–1.39)	0.45 (0.12–1.71)	0.46 (0.10–1.93)
Capecitabine + sorafenib	0.44 (0.17–1.14)	0.45 (0.14–1.49)	0.44 (0.14–1.54)
Capecitabine + vinorelbine	0.44 (0.15–1.28)	0.47 (0.14–1.62)	0.47 (0.13–1.85)
Paclitaxel + epirubicin	0.35 (0.11–1.16)	0.36 (0.09–1.44)	0.31 (0.07–1.37)
Fluorouracil + epirubicin + cyclophosphamide	**0.31 (0.14–0.67)**	**0.32 (0.13–0.78)**	**0.31 (0.12–0.87)**
Paclitaxel + capecitabine	**0.3 (0.09–0.98)**	**0.31 (0.08–1.23)**	0.31 (0.07–1.44)
Paclitaxel + epirubicin + capecitabine	**0.29 (0.09–0.95)**	**0.31 (0.08–1.16)**	0.37 (0.08–1.70)
Capecitabine + sunitinib	**0.23 (0.1–0.56)**	**0.24 (0.08–0.71)**	**0.23 (0.08–0.78)**
Cyclophosphamide + methotrexate + 5-fluorouracil	**0.23 (0.1–0.51)**	**0.23 (0.09–0.61)**	**0.23 (0.09–0.68)**
Model fit statistics	Residual deviance = 58.12 vs. 51 DIC = −4.11	Residual deviance = 52.50 vs. 51 DIC = −4.36 Heterogeneity (SD) = 0.11 (0.01–0.26)	Residual deviance = 52.11 vs. 51 DIC = −4.63 Heterogeneity (SD) = 0.11 (0.01–0.26)

Analyses use data from PALOMA-3 [6]. Statistically significant differences are shown in ***bold***. Endocrine therapies have been excluded from this table, given that the focus is on chemotherapy agents. For vague priors in the random-effects model, a uniform distribution for between-study variance was assumed, as recommended by the National Institute for Health and Care Excellence [9]. Informative priors were based on an estimate of between-study variance using data from previous Cochrane systematic reviews [12]

*CrI* credible interval, *DIC* deviance information criterion, *HR* hazard ratio, *SD* standard deviation

Model fit statistics from the fixed-effects model indicated a poor fit; a total residual deviance value greater than the number of unconstrained data points was obtained (i.e., 58.12 vs. 51). This high residual deviance was largely driven by one study [20], which was removed in a sensitivity analysis and model fit improved (Online Appendix E).

In the second-line random-effects model using vague priors, palbociclib + fulvestrant showed statistically significant improvements versus capecitabine [intermittent: HR 0.29 (95% CrIR 0.10–0.81); continuous: HR 0.25 (0.09–0.70)], mitoxantrone [HR 0.26 (0.11–0.60)], and pegylated liposomal doxorubicin [HR 0.2 (0.06–0.64)] and trended toward improvements versus paclitaxel [HR 0.49 (0.14–1.74)], docetaxel [HR 0.69 (0.2–2.57)], and other monotherapy or combination chemotherapy agents (HRs ranging from 0.23 to 0.89). Similar results and statistical significance were obtained from the random-effects model using informative priors. Model fit statistics were favorable from both random-effects models (Table 2).

### Sensitivity analyses

Sensitivity analyses were conducted to include both the palbociclib phase 2 and 3 studies [5, 15], and to adjust for heterogeneity in median PFS/TTP values (Online Appendix E). For each sensitivity analysis in the first line of therapy, palbociclib + letrozole was associated with improved PFS/TTP relative to all other treatments. After adjusting for heterogeneity in median PFS/TTP values in the second-line analysis, palbociclib + fulvestrant was associated with improved PFS/TTP relative to all chemotherapy comparators. Model fit was favorable across all sensitivity analyses.

## Discussion

An SLR and NMAs were conducted to indirectly compare palbociclib + letrozole and palbociclib + fulvestrant with chemotherapy agents used in the first- and second-line treatment of postmenopausal women with HR +/HER2− ABC/MBC.

The first-line NMA results suggest that palbociclib + letrozole is associated with improved PFS/TTP relative to all other treatments. In the fixed-effects model, statistically significant improvements in PFS/TTP were observed in favor of palbociclib + letrozole relative to capecitabine (intermittent) and mitoxantrone, and trended toward improvements versus paclitaxel, docetaxel, and other monotherapy or combination chemotherapy agents. Findings from the random-effects models suggest that palbociclib + letrozole is associated with improved PFS/TTP relative to all other treatments, although not statistically significant.

The second-line NMA results suggest that palbociclib + fulvestrant is associated with improved PFS/TTP relative to all other chemotherapy treatments. In the fixed-effects model, statistically significant improvements in PFS/TTP were observed in favor of palbociclib + fulvestrant relative to capecitabine (intermittent and continuous), mitoxantrone, and pegylated liposomal doxorubicin, and trended toward improvements versus paclitaxel, docetaxel, and other monotherapy or combination chemotherapy agents. Results from the random-effects models aligned closely with those of the fixed-effects model.

### Strengths and limitations

Palbociclib is a relatively new targeted therapy with the Palbociclib Clinical Trial Development Program still ongoing, and it is currently the only CDK inhibitor approved for use in the US. Since direct head-to-head comparisons have not been made between palbociclib and chemotherapy agents, the current NMA sought to indirectly compare these therapies. To the best of our knowledge, this is the most up-to-date systematic review and NMA to synthesize data for this population of patients with HR+/HER2− ABC/MBC. Notably, analyses were stratified by first and second line of therapy rather than considering both populations simultaneously, as was done in the NMA by Generali et al. [7]. Combining first- and second-line therapies likely violates the exchangeability assumption [13], whereas the current stratified approach adheres to best practices for the conduct of NMA [9, 21]. This study also adheres to PRISMA reporting guidelines (Online Appendix F) [22]. Thorough sensitivity analyses were conducted and yielded similar findings for both the first and second lines of therapy, providing evidence for the robustness of study results.

However, there are a few limitations associated with the analyses employed. Firstly, there is heterogeneity in patient and study characteristics, introduced primarily by the fact that the included studies span several decades. The studies included in our analyses were published between 1992 and 2016, so there is likely some heterogeneity in the diagnostic procedures that were used. Stage migration via technology may result in more patients being diagnosed with advanced stages of disease in more recent trials, which may bias survival rates. However, the structure of the evidence networks limited our ability to adjust for these factors. Despite these issues, considerable effort was taken to account for heterogeneity and inconsistency using best practices [21, 22] and approaches that are analogous to or exceed those employed by other HTA bodies [23, 24]. Various sensitivity analyses were performed, all of which yielded similar findings to the main analyses. Secondly, although analyses were stratified by line of therapy, some connections had to be forced to maintain a connected network. For example, the study by Bachelot et al. [25] was classified as a second-line study, but it was also forced into first-line networks so that chemotherapy agents of interest, such as docetaxel and paclitaxel, could be included. Three studies were also forced into networks based on patient characteristics: Ackland et al. [17], Dixon et al. [19], and Paridaens et al. [18]. Although the study by Dixon et al. [19] is the oldest study included in our analyses, it was also included by Generali et al. [7] and it appears to be the only appropriate trial available that directly compares an endocrine therapy with a chemotherapy. However, only 20–27% of patients in this study were estrogen receptor-positive (ER+), and several key study design characteristics were not reported, including randomization technique, concealment of treatment allocation, or blinding of participants and outcome assessors. Therefore, there is an elevated risk of bias associated with this trial.

Network meta-analyses were also conducted for overall survival; however, these results have been excluded from the current analysis due to immature data in the palbociclib clinical trials.

## Conclusions

Palbociclib + letrozole and palbociclib + fulvestrant demonstrate trends in incremental efficacy compared with chemotherapy agents for the first- and second-line treatment of postmenopausal HR+/HER2− ABC/MBC. Both palbociclib combination therapies consistently showed statistically significant improvements in PFS/TTP versus capecitabine and mitoxantrone, and trended toward improvements versus paclitaxel, docetaxel, and other monotherapy or combination chemotherapy agents. Findings from network meta-analyses were robust to sensitivity analyses, lending credibility to the analyses and conclusions.

## Electronic supplementary material

Below is the link to the electronic supplementary material.

Supplementary material 1 (PDF 1423 kb)

## Abbreviations

ABC: Advanced breast cancer
HER2−: Human epidermal growth factor receptor type 2 negative
HR+: Hormone receptor positive
MBC: Metastatic breast cancer
NMA: Network meta-analysis
PFS: Progression-free survival
RCT: Randomized control trial
TTP: Time to progression
