# Supplementary material for: Systematic review and network meta-analysis comparing palbociclib with chemotherapy agents for the treatment of postmenopausal women with HR-positive and HER2-negative advanced/metastatic breast cancer
Source: Breast Cancer Res Treat. 2017 Jul 27;166(1):167–77. doi: 10.1007/s10549-017-4404-4 (PMC5645434; doi:10.1007/s10549-017-4404-4)
Supplement: Supplementary file 1 — Supplementary material 1 (PDF 1423 kb) [file 10549_2017_4404_MOESM1_ESM.pdf]

SUPPLEMENTARY APPENDICES

Systematic Review and Network Meta-analysis Comparing Palbociclib with Chemotherapy Agents for the Treatment of Postmenopausal Women with HR-Positive and HER2-Negative Advanced/Metastatic Breast Cancer

Authors:
Wilson, F.R., Varu, A., Mitra, D., Cameron, C., Iyer, S.

Corresponding Author:
Chris Cameron
Cornerstone Research Group Inc.
Suite 204, 3228 South Service Road,
Burlington, Ontario,
L7N 3H8
Canada
Phone: 613-852-2374
Email: ccameron@cornerstone-research.com

Appendix A: Search Strategy

**OVERVIEW**
Databases:<br>Ovid MEDLINE<br>Pubmed<br>OVID EMBASE<br>OVID EBM Reviews (Cochrane Library) including:<br>• Cochrane Central Register of Controlled Trials (CENTRAL)<br><br>Search syntax has been customized for each database.<br><br>Date of Search: March 2- 4, 2016<br>Study Types: Randomized Controlled Trials (RCTs)<br>Limits: Studies published after 2014<br><br>Note:<br>†† "*", "# ", and "?" are truncation characters that retrieve all possible suffix variations of the root word e.g. surg* retrieves surgery, surgical, surgeon, etc.

Database	Date Searched	Search Strategy		
**Ovid Medline** In-Process & Other Non-Indexed Citations and **Ovid MEDLINE**(R) 1946 to Present	March 4, 2016<br><br>**Results 570**<br><br>record # 1- 517 & 2542 - 2558	1	exp Breast Neoplasms/	236278
		2	carcinoma, lobular/	4465
		3	exp carcinoma, intraductal, noninfiltrating/	8643
		4	"neoplasms, ductal, lobular, and medullary"/	69
		5	exp paget's disease, mammary/	681
		6	(paget* and (breast* or mammary or nipple*)).tw.	1063
		7	((breast* or mammar*) adj5 (cancer* or neoplasm* or tumo?r* or oncolog* or malignan* or carcinoma* or sarcoma* or adenocarcinoma* or leiomyosarcoma* or dcis or infiltrat* or medullary or tubular)).mp.	325908
		8	((lobul* or duct* or intraduct*) adj5 (cancer* or neoplasm* or tumo?r* or oncolog* or malignan* or carcin* or sarcoma* or adenocarcinoma* or leiomyosarcoma*)).mp.	57676
		9	or/1-8	355772
		10	exp Neoplasm Metastasis/	167751
		11	metasta*.tw.	363575
		12	(advanc* or second* or recurr* or inoperab* or disseminat* or incur*).mp.	2431595
		13	((stage or grade or type) adj3 ("3" or "4" or c or d or iii* or iv*)).mp.	293746
		14	(N1 or N2* or N3* or pN1* or pN2* or pN3*).mp.	154534
		15	or/10-14	3038926
		16	9 and 15	126058
		17	exp Antineoplastic Agents/	893376
		18	antineoplastic protocols/ or antineoplastic combined chemotherapy protocols/	114570
		19	chemotherap*.mp.	366540
		20	((drug* or agent* or therap* or treatment*) adj3 (antineoplastic* or cytotoxic*)).mp.	279740
		21	exp Taxoids/	28643
		22	(taxane* or taxoid*).mp.	14387
		23	paclitaxel/ or albumin-bound paclitaxel/	21205
		24	(Paclitaxel or Taxol or Abraxane).mp.	29773
		25	(Docetaxel or Taxotere).mp.	11909
		26	exp Anthracyclines/	57192
		27	anthracycline*.mp.	12399
		28	exp Doxorubicin/	46160

		29	(Doxorubicin or Adriamycin or Doxil).mp.	57852
		30	Epirubicin/	4579
		31	(Epirubicin or Ellence).mp.	6154
		32	Mitoxantrone/	3922
		33	(Mitoxantrone or Novantrone).mp.	5565
		34	exp Antimetabolites, Antineoplastic/	134097
		35	Antimetabolite*.mp.	27597
		36	exp Fluorouracil/	40504
		37	(Fluorouracil or 5-fluorouracil, or 5-FU or Adrucil).mp.	48157
		38	(Gemcitabine or Gemzar).mp.	12175
		39	Methotrexate/	33518
		40	(Methotrexate or Mexate or Folex or Rheumatrex).mp.	45872
		41	Capecitabine/	3234
		42	(Capecitabine or Xeloda).mp.	4892
		43	exp Alkylating Agents/	117188
		44	Alkylating Agent*.mp.	14776
		45	exp Cyclophosphamide/	48958
		46	(Cyclophosphamide or Cytoxan).mp.	62071
		47	exp Tubulin Modulators/	77359
		48	(Microtubul* or Antimicrotubular* or Tubulin).mp.	79310
		49	(Eribulin or Halaven).mp.	265
		50	Epothilones/	756
		51	Epothilone*.mp.	982
		52	(Ixabepilone or Ixempra).mp.	375
		53	exp Antibiotics, Antineoplastic/	135186
		54	(antibiotic* adj2 (antitumo*r or antineoplastic or cytotoxic)).mp.	23528
		55	Mitomycin/	11152
		56	(Mutamycin or Mitomycin).mp.	18273
		57	exp Vinca Alkaloids/	33625
		58	(Vinorelbine or Navelbine).mp.	3555
		59	exp Aromatase Inhibitors/	6364
		60	aromatase inhibitor*.mp.	7234
		61	(Letrozole or Femara).mp.	2263
		62	(Anastrozole or Arimidex).mp.	1814
		63	(Exemestane or Aromasin).mp.	1094
		64	Everolimus/	2726
		65	((MTOR or mammalian target of rapamycin) adj3 inhibitor*).mp.	5722
		66	(Everolimus or Afinitor or Zortress).mp.	4227
		67	exp Selective Estrogen Receptor Modulators/	24966
		68	Selective estrogen receptor modulator*.mp.	4702
		69	exp Tamoxifen/	18895
		70	(Tamoxifen or Soltamox or Nolvadex).mp.	23395
		71	exp Estrogen Antagonists/	34070
		72	Estrogen Antagonist*.mp.	8049
		73	(Fulvestrant or Faslodex).mp.	2357
		74	exp Cyclin-Dependent Kinases/ai [Antagonists & Inhibitors]	3770
		75	(cyclin dependent kinase* adj3 inhibitor*).mp.	24148
		76	(Palbociclib or Ibrance).mp.	151
		77	exp Progestins/	63478
		78	(progestin* or progestrogen* or progestagen* or antiestrogen* or gestagen*).mp.	27656
		79	Megestrol Acetate/	815
		80	(Megestrol acetate or Megace).mp.	1424
		81	Bendamustine Hydrochloride/	429
		82	(Bendamustine or Treanda or Levact or Ribomustin).mp.	682
		83	Antibodies, Monoclonal/	172191
		84	exp Vascular Endothelial Growth Factors/ai [Antagonists & Inhibitors]	5843
		85	exp Protein Kinase Inhibitors/	59627
		86	exp Protein-Tyrosine Kinases/ai [Antagonists & Inhibitors]	28643

		87	monoclonal antibod*.mp.	167315
		88	((antagonist* or inhibitor*) adj3 (tyrosine or vascular endothelial growth factor* or VEGF or angiogenesis)).mp.	44551
		89	(anti VEGF or antiVEGF).mp.	3601
		90	Bevacizumab/	7860
		91	(Bevacizumab or Avastin).mp.	11982
		92	(Sorafenib or Nexavar).mp.	5191
		93	(Vandetanib or Caprelsa).mp.	453
		94	(Sunitinib or Sutent).mp.	4099
		95	Carboplatin/	9711
		96	(Carboplatin or Paraplatin).mp.	13961
		97	or/17-96	1561145
		98	16 and 97	44643
		99	"randomized controlled trial".pt.	408342
		100	(random* or placebo* or single blind* or double blind* or triple blind*).ti,ab.	893683
		101	(retraction of publication or retracted publication).pt.	8577
		102	99 or 100 or 101	987433
		103	(animals not humans).sh.	4161827
		104	((comment or editorial or meta-analysis or practice-guideline or review or letter or journal correspondence) not "randomized controlled trial").pt.	3544553
		105	(random sampl$ or random digit$ or random effect$ or random survey or random regression).ti,ab. not "randomized controlled trial".pt.	56813
		106	102 not (103 or 104 or 105)	732467
		107	98 and 106	4532
		108	limit 107 to yr="2014 -Current"	570
**Pubmed**	March 2, 2016<br><br>638 results<br><br>518-1138		Search (#26 NOT medline[sb]) Filters: Publication date from 2014/01/01 to 2016/12/31	
			Search (#16 AND #20 AND #24) Filters: Publication date from 2014/01/01 to 2016/12/31	
			Search (#16 AND #20 AND #24)	
			Search (#22 NOT #23)	
			Search (animals [mh] NOT humans [mh])	
			Search ((((((groups [tiab]) OR trial [tiab]) OR randomly [tiab]) OR drug therapy [sh]) OR placebo [tiab]) OR randomized [tiab]) OR controlled clinical trial [pt]) OR randomized controlled trial [pt]	
			Search (#17 OR #18 OR #19)	
			Search (((antiestrogen* or gestagen* or Carboplatin or Paraplatin or Sunitinib or Sutent or Vandetanib or Caprelsa or Sorafenib or Nexavar or Bevacizumab or Avastin or anti VEGF or antiVEGF or monoclonal antibod* or Bendamustine or Treanda or Levact or Ribomustin or Megestrol or Megace or progestin* or progestrogen* or progestagen* or Palbociclib or Ibrance or fulvestrant or faslodex or estrogen antagonist* or Tamoxifen or Soltamox or Nolvadex or selective estrogen receptor modulator* or Everolimus or Afinitor or Zortress or rapamycin inhibitor* or MTOR inhibitor* or Exemestane or Aromasin or Anastrozole or Arimidex or Letrozole or Femara or aromatase inhibitor* or Vinorelbine or Navelbine or Vinca Alkaloid* or Mutamycin or Mitomycin or Ixabepilone or Ixempra or Epothilone* or Eribulin or Halaven or Microtubul* or Antimicrotubular* or Tubulin or Cyclophosphamide or Cytoxan or Alkylating Agent* or Capecitabine or Xeloda or Methotrexate or Mexate or Folex or Rheumatrex or Gemcitabine or Gemzar or Fluorouracil or 5-fluorouracil or 5-FU or Adrucil or Antimetabolite* or Mitoxantrone or Novantrone or Epirubicin or Ellence or Doxorubicin or Adriamycin or Doxil or anthracycline* or Docetaxel or Taxotere or Paclitaxel or Taxol or Abraxane or taxane* or taxoid*)) OR ((antagonist* or inhibitor*) AND (tyrosine or vascular endothelial growth factor* or VEGF or angiogenesis)))) OR (cyclin dependent kinase* AND inhibitor*)) OR (antibiotic* AND (antitumor or antitumour or antineoplastic or cytotoxic)))	
			Search chemotherap*	
			Search ((drug* or agent* or therap* or treatment* or protocol*) AND (antineoplastic* or cytotoxic*))	
			Search (#15 AND #9)	
			Search (#10 OR #11 OR #12 OR #13 OR #14)	
			Search (N1 or N2* or N3* or pN1* or pN2* or pN3*)	
			Search ((stage or grade or type) AND ("3" or "4" or c or d or iii* or iv*))	
			Search (advanc* or second* or recurr* or inoperab* or disseminat* or incur*)	
			Search metasta*	

		Search Neoplasm Metastasis[MeSH Terms]<br>Search ((#1 OR #2 OR #3 OR #4 OR #5 OR #6 OR #8))<br>Search (((breast* or mammar*) AND (cancer* or neoplasm* or tumour* or tumor* or oncolog* or malignan* or carcinoma* or sarcoma* or adenocarcinoma* or leiomyosarcoma* or dcis or duct* or infiltrat* or intraduct* or lobul* or medullary or tubular)))<br>Search ((paget* AND (breast* OR mammary OR nipple*)))<br>Search paget's disease, mammary[MeSH Terms]<br>Search "neoplasms, ductal, lobular, and medullary"[MeSH Terms]<br>Search carcinoma, intraductal, noninfiltrating[MeSH Terms]<br>Search carcinoma, lobular[MeSH Terms]<br>Search Breast Neoplasms[MeSH Terms]
**OVID EMBASE**	March 4, 2016<br><br>975 results<br><br>record # 1139-1994<br><br>& 2559-2600	1 exp *breast tumor/ 236202<br>2 *breast cancer/ 157207<br>3 *basal like breast cancer/ or *breast adenocarcinoma/ or *breast carcinogenesis/ or *breast carcinoma/ or *breast sarcoma/ or exp *estrogen receptor positive breast cancer/ or *"hereditary breast and ovarian cancer syndrome"/ or *inflammatory breast cancer/ or *intraductal carcinoma/ or *paget nipple disease/ or *progesterone receptor positive breast cancer/ 37272<br>4 (paget* and (breast* or mammary or nipple*)).tw. 1072<br>5 ((breast* or mammar*) adj5 (cancer* or neoplasm* or tumo?r* or oncolog* or malignan* or carcinoma* or sarcoma* or adenocarcinoma* or leiomyosarcoma* or dcis or infiltrat* or medullary or tubular)).mp. 414137<br>6 ((lobul* or duct* or intraduct*) adj5 (cancer* or neoplasm* or tumo?r* or oncolog* or malignan* or carcin* or sarcoma* or adenocarcinoma* or leiomyosarcoma*)).mp. 67534<br>7 or/1-6 455770<br>8 *metastasis/ 38534<br>9 metasta*.tw. 452114<br>10 (advanc* or second* or recurr* or inoperab* or disseminat* or incur*).mp. 2775188<br>11 ((stage or grade or type) adj3 ("3" or "4" or c or d or iii* or iv*)).mp. 351190<br>12 (N1 or N2* or N3* or pN1* or pN2* or pN3*).mp. 259462<br>13 or/8-12 3453702<br>14 7 and 13 175151<br>15 *breast metastasis/ 4687<br>16 14 or 15 175298<br>17 exp *antineoplastic agent/ 602681<br>18 exp *chemotherapy/ 93876<br>19 chemotherap*.mp. 545008<br>20 ((drug* or agent* or therap* or treatment* or protocol*) adj3 (antineoplastic* or cytotoxic*)).mp. 277990<br>21 (taxane* or taxoid*).mp. 21401<br>22 (Paclitaxel or Taxol or Abraxane).mp. 81330<br>23 (Docetaxel or Taxotere).mp. 42750<br>24 anthracycline*.mp. 28324<br>25 (Doxorubicin or Adriamycin or Doxil).mp. 135142<br>26 (Epirubicin or Ellence).mp. 23719<br>27 (Mitoxantrone or Novantrone).mp. 20033<br>28 Antimetabolite*.mp. 8575<br>29 (Fluorouracil or 5-fluorouracil or 5-FU or Adrucil).mp. 102735<br>30 (Gemcitabine or Gemzar).mp. 40999<br>31 (Methotrexate or Mexate or Folex or Rheumatrex).mp. 124507<br>32 (Capecitabine or Xeloda).mp. 21195<br>33 Alkylating Agent*.mp. 16712<br>34 (Cyclophosphamide or Cytoxan).mp. 152370<br>35 (Microtubul* or Antimicrotubular* or Tubulin).mp. 77563<br>36 (Eribulin or Halaven).mp. 1033<br>37 Epothilone*.mp. 2086<br>38 (Ixabepilone or Ixempra).mp. 1565<br>39 (antibiotic* adj2 (antitumo?r or antineoplastic or cytotoxic)).mp. 5777<br>40 (Mutamycin or Mitomycin).mp. 32659

		41	(Vinorelbine or Navelbine).mp.	15070
		42	aromatase inhibitor*.mp.	13587
		43	(Letrozole or Femara).mp.	8590
		44	(Anastrozole or Arimidex).mp.	7743
		45	(Exemestane or Aromasin).mp.	4768
		46	MTOR inhibitor*.mp.	8071
		47	mammalian target of rapamycin inhibitor*.mp.	8383
		48	(Everolimus or Afinitor or Zortress).mp.	18512
		49	Selective estrogen receptor modulator*.mp.	7824
		50	(Tamoxifen or Soltamox or Nolvadex).mp.	50959
		51	Estrogen Antagonist*.mp.	751
		52	(Fulvestrant or Faslodex).mp.	6317
		53	histone deacetylase inhibitor*.mp.	15470
		54	(cyclin dependent kinase* adj3 inhibitor*).mp.	24106
		55	(Palbociclib or Ibrance).mp.	526
		56	(progestin* or progestrogen* or progestagen*).mp.	11861
		57	(Megestrol or Megace).mp.	4792
		58	(Bendamustine or Treanda or Levact or Ribomustin).mp.	3480
		59	monoclonal antibod*.mp.	234578
		60	((antagonist* or inhibitor*) adj2 (tyrosine or vascular endothelial growth factor* or VEGF or angiogenesis)).mp.	59723
		61	(anti VEGF or antiVEGF).mp.	5534
		62	(Bevacizumab or Avastin).mp.	40222
		63	(Sorafenib or Nexavar).mp.	19746
		64	(Vandetanib or Caprelsa).mp.	3545
		65	(Sunitinib or Sutent).mp.	16336
		66	(Carboplatin or Paraplatin).mp.	52432
		67	antiestrogen*.mp.	10796
		68	gestagen*.mp.	19283
		69	or/17-68	1598388
		70	16 and 69	78983
		71	random*.ti,ab.	1008885
		72	factorial*.ti,ab.	25172
		73	(crossover* or cross over*).ti,ab.	70814
		74	((doubl* or singl*) adj blind*).ti,ab.	155779
		75	(assign* or allocat* or volunteer* or placebo*).ti,ab.	698087
		76	crossover procedure/	46186
		77	double blind procedure/	119534
		78	single blind procedure/	21588
		79	randomized controlled trial/	379496
		80	71 or 72 or 73 or 74 or 75 or 76 or 77 or 78 or 79	1548071
		81	animal/ not human/	675529
		82	nonhuman/	4211541
		83	exp animal experiment/	1405919
		84	exp experimental animal/	448483
		85	animal model/	829126
		86	exp rodent/	2317514
		87	(rat or rats or mouse or mice).ti.	947238
		88	81 or 82 or 83 or 84 or 85 or 86 or 87	5293256
		89	80 not 88	1306055
		90	70 and 89	10550
		91	limit 90 to yr="2014 -Current"	1819
		92	limit 91 to (conference abstract or conference paper or conference proceeding or "conference review")	844
		93	91 not 92	975

**EBM Reviews:**<br><br>**CENTRAL**	March 4, 2016<br><br>685 results<br><br>record # 1995-2541	1	(paget* and (breast* or mammary or nipple*)).ti,ab,kw,sh.	15
		2	((breast* or mammar*) adj5 (cancer* or neoplasm* or tumo?r* or oncolog* or malignan* or carcin* or sarcoma* or adenocarcinoma* or leiomyosarcoma* or dcis or infiltrat* or medullary or tubular)).ti,ab,kw,sh.	18102
		3	((lobul* or duct* or intraduct*) adj5 (cancer* or neoplasm* or tumo?r* or oncolog* or malignan* or carcin* or sarcoma* or adenocarcinoma* or leiomyosarcoma*)).ti,ab,kw,sh.	693
		4	1 or 2 or 3	18435
		5	metasta*.ti,ab,kw,sh.	14598
		6	(advanc* or second* or recurr* or inoperab* or disseminat* or incur*).ti,ab,kw,sh.	145795
		7	((stage or grade or type) adj3 ("3" or "4" or c or d or iii* or iv*)).ti,ab,kw,sh.	20252
		8	(N1 or N2* or N3* or pN1* or pN2* or pN3*).ti,ab,kw,sh.	20905
		9	or/5-8	178741
		10	chemotherap*.ti,ab,kw,sh.	30860
		11	((drug* or agent* or therap* or treatment* or protocol*) adj3 (antineoplastic* or cytotoxic*)).ti,ab,kw,sh.	10042
		12	(taxane* or taxoid*).ti,ab,kw,sh.	1588
		13	(Paclitaxel or Taxol or Abraxane).ti,ab,kw,sh.	3688
		14	(Docetaxel or Taxotere).ti,ab,kw,sh.	2572
		15	anthracycline*.ti,ab,kw,sh.	1277
		16	(Doxorubicin or Adriamycin or Doxil).ti,ab,kw,sh.	5282
		17	(Epirubicin or Ellence).ti,ab,kw,sh.	1818
		18	(Mitoxantrone or Novantrone).ti,ab,kw,sh.	875
		19	Antimetabolite*.ti,ab,kw,sh.	1159
		20	(Fluorouracil or 5-fluorouracil or 5-FU or Adrucil).ti,ab,kw,sh.	7059
		21	(Gemcitabine or Gemzar).ti,ab,kw,sh.	1904
		22	(Methotrexate or Mexate or Folex or Rheumatrex).ti,ab,kw,sh.	5589
		23	(Capecitabine or Xeloda).ti,ab,kw,sh.	1168
		24	Alkylating Agent*.ti,ab,kw,sh.	360
		25	(Cyclophosphamide or Cytoxan).ti,ab,kw,sh.	7068
		26	(Microtubul* or Antimicrotubular* or Tubulin).ti,ab,kw,sh.	162
		27	(Eribulin or Halaven).ti,ab,kw,sh.	48
		28	Epothilone*.ti,ab,kw,sh.	52
		29	(Ixabepilone or Ixempra).ti,ab,kw,sh.	62
		30	(antibiotic* adj2 (antitumo?r or antineoplastic or cytotoxic)).ti,ab,kw,sh.	29
		31	(Mutamycin or Mitomycin).ti,ab,kw,sh.	1994
		32	(Vinorelbine or Navelbine).ti,ab,kw,sh.	803
		33	aromatase inhibitor*.ti,ab,kw,sh.	945
		34	(Letrozole or Femara).ti,ab,kw,sh.	719
		35	(Anastrozole or Arimidex).ti,ab,kw,sh.	600
		36	(Exemestane or Aromasin).ti,ab,kw,sh.	392
		37	MTOR inhibitor*.ti,ab,kw,sh.	216
		38	mammalian target of rapamycin inhibitor*.ti,ab,kw,sh.	159
		39	(Everolimus or Afinitor or Zortress).ti,ab,kw,sh.	1221
		40	Selective estrogen receptor modulator*.ti,ab,kw,sh.	488
		41	(Tamoxifen or Soltamox or Nolvadex).ti,ab,kw,sh.	3365
		42	Estrogen Antagonist*.ti,ab,kw,sh.	360
		43	(Fulvestrant or Faslodex).ti,ab,kw,sh.	160
		44	histone deacetylase inhibitor*.ti,ab,kw,sh.	69
		45	(cyclin dependent kinase* adj3 inhibitor*).ti,ab,kw,sh.	34
		46	(Palbociclib or Ibrance).ti,ab,kw,sh.	16
		47	(progestin* or progestrogen* or progestagen*).ti,ab,kw,sh.	1347
		48	(Megestrol or Megace).ti,ab,kw,sh.	426
		49	(Bendamustine or Treanda or Levact or Ribomustin).ti,ab,kw,sh.	81
		50	monoclonal antibod*.ti,ab,kw,sh.	3027
		51	((antagonist* or inhibitor*) adj2 (tyrosine or vascular endothelial growth factor* or VEGF or angiogenesis)).ti,ab,kw,sh.	899
		52	(anti VEGF or antiVEGF).ti,ab,kw,sh.	210
		53	(Bevacizumab or Avastin).ti,ab,kw,sh.	1581

		54	(Sorafenib or Nexavar).ti,ab,kw,sh.	435
		55	(Vandetanib or Caprelsa).ti,ab,kw,sh.	67
		56	(Sunitinib or Sutent).ti,ab,kw,sh.	306
		57	(Carboplatin or Paraplatin).ti,ab,kw,sh.	2702
		58	antiestrogen*.ti,ab,kw,sh.	280
		59	gestagen*.ti,ab,kw,sh.	231
		60	or/10-59	57958
		61	4 and 9 and 60	6624
		62	limit 61 to yr="2014 -Current"	685

Appendix B: PICOS Eligibility Criteria

Table 1: Summary of PICOS Eligibility Criteria for the Systematic Review and NMA

Item	Description
**Population**	Postmenopausal women with HR+/HER2- ABC/MBC receiving first- or second-line therapy for their disease.
**Intervention/ Index Node**	• **First-line**: Palbociclib 125 mg daily (3 weeks on and 1 week off) + Letrozole 2.5 mg daily<br>• **Second-line**: Palbociclib 125 mg daily (3 weeks on and 1 week off) + Fulvestrant 500 mg (every 14 days for first 3 injections, then every 28 days)
**Comparators****	Endocrine-based therapies, chemotherapy agents, and/or chemotherapy agents + biological therapies:<br>• **First-line**: anastrozole, bevacizumab, capecitabine, carboplatin*, cyclophosphamide, docetaxel, epirubicin, everolimus, exemestane, fluorouracil, gemcitabine, letrozole, liposomal doxorubicin, megestrol acetate*, methotrexate, mitoxantrone*, paclitaxel*, sunitinib, tamoxifen, vinorelbine<br>• **Second-line**: aminoglutethimide, anastrozole, bevacizumab, capecitabine, carboplatin, corticosteroid, cyclophosphamide, docetaxel, doxorubicin, epirubicin, everolimus, exemestane, fluorouracil, fulvestrant, gemcitabine, ixabepilone, letrozole, megestrol acetate, methotrexate, mitoxantrone, motesanib, nab-paclitaxel, paclitaxel, pegylated liposomal doxorubicin, sorafenib, sunitinib, vinorelbine<br>• Bendamustine hydrochloride, mutamycin, and vandetanib were also considered as comparators, but did not end up being included in final evidence networks<br>• Any combination of the above therapies
**Outcomes**	• PFS/TTP
**Study design**	• Randomized, controlled, prospective clinical trials (phase 2 and 3 RCTs)<br>• Abstracts (e.g., conference abstracts)

ABC/MBC = advanced/metastatic breast cancer; ER+ = estrogen receptor-positive; HER2- = human epidermal growth factor receptor 2-negative; HR+ = hormone receptor-positive; PFS = progression-free survival; RCT = randomized controlled trial; TTP = time to progression.

*These therapies were included in first-line networks as forced connections based on line of therapy.

**Comparators are separated by line of therapy based on the evidence networks used in the primary analyses.

Appendix C: Summary of Study and Patient Baseline Characteristics and Prior Therapies

Table 2: Summary of Study and Patient Characteristics Included in Network Meta-Analysis for First-Line PFS/TTP

Study	Treatments	Blinding	Analysis Population	FUP (median months)	N total	Median age (yrs)	Post-menopausal (%)	ER+ (%)	PgR+ (%)	HR+ (%)	HER2- (%)	Visceral metastases (%)
**Bonneterre 2004 [1]**	DOC + EPI	OL	ITT	23.8	70	54	NR	54	51	NR	NR	NR
	FEC	OL	ITT	23.8	72	54	NR	67	60	NR	NR	NR
**Cinieri 2014 [2]**	CAP + VIN	OL	ITT	NR	49	58	NR	NR	NR	61	100	80
	PAC + GEM	OL	ITT	NR	50	56	NR	NR	NR	50	100	82
	DOC + GEM	OL	ITT	NR	50	57	NR	NR	NR	66	100	74
**Finn 2015** [3]**	PAL + LET 2.5	OL	ITT	29.6	84	63	100	100	NR	100	100	44
	LET 2.5	OL	ITT	27.9	81	64	100	100	NR	100	100	53
**Finn 2016 [4]**	PAL + LET 2.5	DB	ITT	23	666	62	100	100	NR	100	100	49.3
	LET 2.5	DB	ITT	23		62	100	100	NR	100	100	
**Miles 2010 [5]**	DOC	DB	ITT	25	241	55	NR	NR	NR	78	100	NR
	DOC + BEVA 7.5	DB	ITT	25	248	54	NR	NR	NR	78	100	NR
	DOC + BEVA 15	DB	ITT	25	247	55	NR	NR	NR	76	100	NR
**Mouridsen 2001 [6]**	LET 2.5	DB	ITT	18	453	65	100	NR	NR	65	NR	43
	TAM	DB	ITT	18	454	64	100	NR	NR	67	NR	46
**Nabholtz 2000 [7]**	ANA 1	DB	ITT	17.7	171	68	100	84.8	67.3	88.3	NR	48.5
	TAM	DB	ITT	17.7	182	67	100	85.7	69.8	88.9	NR	47.8
**O'Shaughnessy 2001 [8]**	CAP	OL	mITT	NR	62	69	100	NR	NR	NR	NR	NR
	CMF	OL	mITT	NR	33	70	100	NR	NR	NR	NR	NR
**Paridaens 2008 [9]**	EXE	OL	ITT	29	182	63	100	87.9	61.5	92.3	NR	47.8
	TAM	OL	ITT	29	189	62	100	89.4	60.8	94.2	NR	46.6
**Robert 2011a [10]**	PAC + SUN	OL	ITT	8.1	242	57	NR	NR	NR	76	97	NR
	PAC + BEVA	OL	ITT	8.1	243	57	NR	NR	NR	76	97	NR
**Robert 2011b [11]**	CAP	DB	ITT	15.6	206	57	NR	NR	NR	73.7	95.1	71.4
	CAP + BEVA	DB	ITT	15.6	409	56	NR	NR	NR	77.4	95.8	67.5
**Robertson 2012** [12]**	FUL 500	OL	ITT	18.8	102	66	Yes	96.1	80.4	100	47.1	47.1
	ANA 1	OL	ITT	12.9	103	68	Yes	97.0	78.6	100	47.6	56.3
**Vici 2011 [13]**	DOC + GEM	NR	ITT	NR	36	61	72.2	NR	NR	69.4	52.8	50
	DOC + CAP	NR	ITT	NR	36	63	77.8	NR	NR	72.2	55.6	50
**Welt 2016 [14]**	CAP + BEVA	OL	mITT	22.2	297	NR	81.8	NR	NR	79.5	99.3	78.1
	CAP + BEVA + VIN	OL	mITT	23.6	295	NR	81.7	NR	NR	79	99.3	76.3
**Yardley 2009 [15]**	LIP DOX	NR	ITT	NR	50	62	NR	78	NR	NR	NR	88
	DOC	NR	ITT	NR	52	63	NR	63	NR	NR	NR	87

**Yardley 2015 [16]**	PAC + BEVA + EVE	NR	ITT	16	56	61	NR	NR	NR	79	100	NR
	PAC + BEVA	NR	ITT	16	57	57	NR	NR	NR	79	100	NR
**Forced Second-line Connections**												
**Bachelot 2011 [17]**	DOC + CAP	NR	ITT	42	33	57	64	85	NR	NR	100	NR
	DOC + EPI	NR	ITT	42	35	59	71	82	NR	NR	100	NR
**Dixon 1992* [18]**	MGA	NR	ITT	NR	30	64	100	27	NR	NR	NR	30
	MITOX	NR	ITT	NR	30	61	100	20	NR	NR	NR	30
**Fountzilas 2009 [19]**	PAC + CARBO	NR	ITT	34	136	60	82	65	NR	NR	57	71
	DOC + GEM	NR	ITT	34	144	60	76	67	NR	NR	56	71
	PAC	NR	ITT	34	136	60.5	79	71	NR	NR	60	76.5
**Ghosn 2011 [20]**	CAP + VIN	OL	ITT	NR	41	51	NR	78	65.9	NR	100	NR
	DOC	OL	ITT	NR	29	57	NR	72.4	62.1	NR	100	NR
**Heidemann 2002 [21]**	MITOX	NR	Efficacy	13.6	119	NR	NR	43	35	48	NR	NR
	FEC	NR	Efficacy	13.6	119	NR	NR	58	48	60	NR	NR
**Kaufmann 2000 [22]**	EXE	DB	ITT	11.3	366	65	100	NR	NR	67.2	NR	56.6
	MGA	DB	ITT	11.3	403	65	100	NR	NR	68	NR	59.3
**Miller 2007 [23]**	PAC + BEVA	OL	ITT	41.6	347	56	NR	59.9	44.7	NR	92.5	79.5
	PAC	OL	ITT	43.5	326	55	NR	62.9	45.1	NR	89.9	87.1
**Forced Key Connections Based on Patient Characteristics**												
**Ackland 2001 [24]**	FEC	NR	ITT	21	223	56	75	17	NR	NR	NR	57
	CMF	NR	ITT	19	237	55	69	14	NR	NR	NR	60

ANA 1 = anastrozole 1 mg; BEVA = bevacizumab; CAP = capecitabine; CARBO = carboplatin; CMF = cyclophosphamide + methotrexate + 5-fluorouracil; DB = double blind; DOC = docetaxel; EPI = epirubicin; ER+ = estrogen receptor-positive; EVE = everolimus; EXE = exemestane; FEC = fluorouracil + epirubicin + cyclophosphamide; FUP = follow-up; GEM = gemcitabine; HER2- = human epidermal growth factor receptor type 2-negative; HR+ = hormone receptor-positive; ITT = intention-to-treat; LET 2.5 = letrozole 2.5 mg; LIP DOX = liposomal doxorubicin; MGA = megestrol acetate; MITOX = mitoxantrone; mITT = modified intention-to-treat; NR = not reported; OL = open label; PAC = paclitaxel; PgR+ = progesterone receptor-positive; SUN = sunitinib; TAM = tamoxifen; VIN = vinorelbine.

* Also a forced key connection based on patient characteristics.

**Included in sensitivity analyses only.

Table 3: Summary of Study and Patient Characteristics Included in Network Meta-Analysis for Second-line PFS/TTP

Study	Treatments	Blinding	Analysis Population	FUP (median months)	N total	Median age (yrs)	Post-menopausal (%)	ER+ (%)	PgR+ (%)	HR+ (%)	HER2- (%)	Visceral metastases (%)
**Bachelot 2011 [17]**	DOC + CAP	NR	ITT	42	33	57	64	85	NR	NR	100	NR
	DOC + EPI	NR	ITT	42	35	59	71	82	NR	NR	100	NR
**Baselga 2012 [25]**	CAP + SOR	DB	ITT	NR	115	Mean 55.1	NR	NR	NR	81.7	100	75.7
	CAP	DB	ITT	NR	114	Mean 54.4	NR	NR	NR	69.3	100	73.7
**Bergh 2012 [26]**	DOC + SUN	OL	ITT	18	296	54	NR	74	64	NR	100	74
	DOC	OL	ITT	18	297	56	NR	70	54	NR	100	70
**Brufsky 2011 [27]**	PAC + BEVA	OL	ITT	14.6	94	57.5	NR	63.8	44.7	NR	92.6	72.3
	PAC + BEVA + GEM	OL	ITT	17.1	93	55.2	NR	72	61.3	NR	92.5	71
**Buzdar 1997 [28]**	ANA 1	DB	ITT	6	128	Mean 65	Yes	86	64	87	NR	40
	MGA	OL	ITT	6	128	Mean 66	Yes	79	57	81	NR	47
**Buzdar 2001 [29]**	MGA	DB	ITT	18	201	65.9	Yes	NR	NR	80	NR	48
	LET 2.5	DB	ITT	18	199	65.5	Yes	NR	NR	80	NR	48
**Campone 2013 [30]**	CAP + VIN	OL	ITT	NR	44	55	NR	65.9	52.3	NR	75	65.9
	DOC + CAP	OL	ITT	NR	48	52.2	NR	58.3	43.8	NR	79.2	64.6
**Chan 2009 [31]**	DOC + GEM	NR	ITT	NR	153	56	NR	NR	NR	69	49	84
	DOC + CAP	NR	ITT	NR	152	53	NR	NR	NR	72	46	88
**Chia 2008 [32]**	FUL 500/250	DB	ITT	NR	351	63	Yes	NR	NR	98.3	NR	56.1
	EXE	DB	ITT	NR	342	63	Yes	NR	NR	98.2	NR	57.9
**Cristofanilli 2016 [33]**	PAL + FUL 500	DB	ITT	8.9	347	57	79.3	NR	NR	68.6	100	59.4 (per protocol)
	FUL 500	DB	ITT	8.9	174	56	79.3	NR	NR	63.8	100	60.3 (per protocol)
**Crown 2013 [34]**	CAP + SUN	OL	ITT	14.3	221	52	NR	66	50	NR	NR	NR
	CAP	OL	ITT	14.3	221	54	NR	68	54	NR	NR	NR
**Del Mastro 2013 [35]**	DOC + GEM	OL	ITT	NR	118	57.3	70.4	70.3	61.0	NR	100	70.3
	PAC + GEM	OL	ITT	NR	123	56.3	68.9	75.7	69.2	NR	100	66
**Di Leo 2010 [36]**	FUL 250	DB	ITT	NR	374	61	Yes	100	71.1	100	NR	62
	FUL 500	DB	ITT	NR	362	61	Yes	100	66.6	100	NR	66
**Dombernowsky 1998 [37]**	MGA	DB	ITT	18-20	189	Mean 64	Yes	NR	NR	58.7	NR	40.7
	LET 2.5	DB	ITT	18-20	174	Mean 63.6	Yes	NR	NR	57.5	NR	42.5
**Fountzilas 2004 [38]**	PAC + EPI	NR	ITT	23.5	163	59	76	55	44	NR	64	68
	PAC + CARBO	NR	ITT	23.5	164	59	74	62	49	NR	64	75
**Fountzilas 2009 [19]**	PAC + CARBO	NR	ITT	34	136	60	82	65	NR	NR	57	71
	DOC + GEM	NR	ITT	34	144	60	76	67	NR	NR	56	71
	PAC	NR	ITT	34	136	60.5	79	71	NR	NR	60	76.5

**Gershanovich 1998 [39]**	AGH + CS	OL	mITT	20	178	65	Yes	NR	NR	50.5	NR	39.9
	LET 2.5	OL	mITT	20	185	66	Yes	NR	NR	60.5	NR	48.6
**Ghosn 2011 [20]**	CAP + VIN	OL	ITT	NR	41	51	NR	78	65.9	NR	100	NR
	DOC	OL	ITT	NR	29	57	NR	72.4	62.1	NR	100	NR
**Hatschek 2012 [40]**	PAC + EPI	OL	ITT	NR	143	57	NR	72	56	NR	92	NR
	PAC + EPI + CAP	OL	ITT	NR	144	55.7	NR	82	61	NR	95	NR
**Heidemann 2002 [21]**	MITOX	NR	Efficacy	13.6	119	NR	NR	43	35	48	NR	NR
	FEC	NR	Efficacy	13.6	119	NR	NR	58	48	60	NR	NR
**Howell 2002 [41]**	FUL 250	OL	ITT	14.4	222	Mean 63	Yes	70.3	38.7	73.4	NR	NR (13.5 visceral only)
	ANA 1	OL	ITT	14.4	229	Mean 64	Yes	75.5	41.5	79.9	NR	NR (17.9 visceral only)
**Jiang 2014 [42]**	FUL 250	DB	NR	NR	110	Mean 53.6	Yes	100	NR	100	NR	NR
	FUL 500	DB	NR	NR	111	Mean 53.1	Yes	100	NR	100	NR	NR
**Jonat 1996 [43]**	MGA	OL	ITT	6	125	Mean 64	Yes	58	41	NR	NR	42
	ANA 1	DB	ITT	6	135	Mean 65	Yes	62	42	NR	NR	54
**Jones 2005 [44]**	DOC	OL	ITT	61.2	225	56	88	51.1	36.4	56	NR	NR
	PAC	OL	ITT	61.2	224	54	86.6	42	35.3	50	NR	NR
**Kaufmann 2000 [22]**	EXE	DB	ITT	11.3	366	65	100	NR	NR	67.2	NR	56.6
	MGA	DB	ITT	11.3	403	65	100	NR	NR	68	NR	59.3
**Luck 2013 [45]**	PAC + CAP	NR	ITT	24.9	170	57	NR	NR	NR	70	NR	56
	PAC + EPI	NR	ITT	24.9	170	58	NR	NR	NR	68	NR	47
**Martin 2011 [46]**	PAC + MOT	DB	ITT	NR	91	55	76	NR	NR	80	100	NR
	PAC	DB	ITT	NR	94	53	66	NR	NR	80	100	NR
	PAC + BEVA	OL	ITT	NR	97	55	64	NR	NR	80	100	NR
**Martin 2015 [47]**	CAP (Intermittent)	NR	ITT	NR	95	61	65.3	NR	NR	79	100	78.1
	CAP (Continuous)	NR	ITT	NR	97	59	61.9	NR	NR	78.4	100	NR
**Miller 2007 [23]**	PAC + BEVA	OL	ITT	41.6	347	56	NR	59.9	44.7	NR	92.5	79.5
	PAC	OL	ITT	43.5	326	55	NR	62.9	45.1	NR	89.9	87.1
**Ohno 2010 [48]**	FUL 250	DB	ITT	NR	45	61	Yes	100	71.7	100	80	57.8
	FUL 500/250	DB	ITT	NR	51	62	Yes	100	70.6	100	90.8	54.9
	FUL 500	DB	ITT	NR	47	61	Yes	100	63.8	100	85.1	57.4
**Osborne 2002 [49]**	FUL 250	DB	ITT	16.8	206	Mean 63	Yes	82.5	62.1	86.9	NR	NR (18.9 visceral only)
	ANA 1	DB	ITT	16.8	194	Mean 62	Yes	80.4	54.6	87.1	NR	NR (23.2 visceral only)
**Papadimitriou 2009 [50]**	DOC	NR	Per-protocol	NR	34	57	NR	NR	NR	74	29	NR
	DOC + GEM	NR	Per-protocol	NR	41	57	NR	NR	NR	66	34	NR
**Pritchard 2010 [51]**	FUL 250	DB	ITT	NR	47	63	Yes	100	63.8	100	78.7	72.3

	FUL 500/250	DB	ITT	NR	51	69	Yes	100	62.7	100	72.5	80.4
	FUL 500	DB	ITT	NR	46	67	Yes	100	69.6	100	69.6	80.4
**Rugo 2013 [52]**	IXA 16 + BEVA	OL	ITT	>19	46	60	NR	80.4	NR	NR	97.8	NR
	IXA 40 + BEVA	OL	ITT	>19	45	59	NR	77.8	NR	NR	100	NR
	PAC + BEVA	OL	ITT	>19	32	59	NR	84.4	NR	NR	100	NR
**Rugo 2015 [53]**	PAC + BEVA	NR	mITT	25	283	NR	NR	NR	NR	71	92	77
	NAB-PAC + BEVA	NR	mITT	25	271	NR	NR	NR	NR	73	93	76
	IXA 16 + BEVA	NR	mITT	25	245	NR	NR	NR	NR	72	93	81
**Smorenburg 2014 [54]**	Peg. LIP DOX	NR	ITT	39	40	NR	NR	62	NR	NR	62	30
	CAP (Intermittent)	NR	ITT	39	38	NR	NR	58	NR	NR	61	32
**Stockler 2011 [55]**	CMF	NR	ITT	39.6	109		NR	NR	NR	64	NR	NR
	CAP (Intermittent)	NR	ITT	39.6	108	62	NR	NR	NR	62	NR	NR
	CAP (Continuous)	NR	ITT	39.6	108		NR	NR	NR	67	NR	NR
**Wang 2015 [56]**	DOC + CAP	OL	ITT	36	104	52	65.7	61.5	60.6	NR	66.3	69.9
	CAP + VIN	OL	ITT	36	102	52	62.6	59.8	60.8	NR	67.6	65.7
**Xu 2011 [57]**	FUL 250	DB	ITT	NR	121	Mean 53.4	Yes	100	NR	NR	NR	NR
	ANA 1	DB	ITT	NR	113	Mean 54.8	Yes	100	NR	NR	NR	NR
**Yardley 2013 [58]**	EVE + EXE	DB	ITT	17.7	485	62	100	100	NR	100	100	58
	EXE	DB	ITT	17.7	239	61	100	100	NR	100	100	59
**Forced First-line Connections**												
**Ackland 2001* [24]**	FEC	NR	ITT	21	223	56	75	17	NR	NR	NR	57
	CMF	NR	ITT	19	237	55	69	14	NR	NR	NR	60
**Bonneterre 2004 [1]**	DOC + EPI	OL	ITT	23.8	70	54	NR	54	51	NR	NR	NR
	FEC	OL	ITT	23.8	72	54	NR	67	60	NR	NR	NR
**Forced Key Connections Based on Patient Characteristics**												
**Dixon 1992 [18]**	MGA	NR	ITT	NR	30	64	100	27	NR	NR	NR	30
	MITOX	NR	ITT	NR	30	61	100	20	NR	NR	NR	30
**Paridaens 2000 [59]**	PAC	NR	ITT	NR	166	54	NR	27 (34% unknown)	NR	NR	NR	78
	DOX	NR	ITT	NR	165	55	NR	24 (37% unknown)	NR	NR	NR	75

AGH = aminoglutethimide; ANA 1 = anastrozole 1 mg; BEVA = bevacizumab; CAP = capecitabine; CARBO = carboplatin; CMF = cyclophosphamide + methotrexate + 5-fluorouracil; CS = corticosteroid; DB = double blind; DOC = docetaxel; DOX = doxorubicin; EPI = epirubicin; ER+ = estrogen receptor-positive; EVE = everolimus; EXE = exemestane; FEC = fluorouracil + epirubicin + cyclophosphamide; FUL 500/250 = fulvestrant 500 mg/250 mg; FUP = follow-up; GEM = gemcitabine; HER2- = human epidermal growth factor receptor type 2-negative; HR+ = hormone receptor-positive; ITT = intention-to-treat; IXA = ixabepilone; LET 2.5 = letrozole 2.5 mg; MGA = megestrol acetate; MITOX = mitoxantrone; mITT = modified intention-to-treat; MOT = motesanib; NAB-PAC = nanoparticle albumin-bound paclitaxel; NR = not reported; OL = open label; PAC = paclitaxel; PAL = palbociclib; Peg. LIP DOX = pegylated liposomal doxorubicin; SOR = sorafenib; SUN = sunitinib; VIN = vinorelbine.

*Also a forced key connection based on patient characteristics.

Appendix D: Risk of Bias Assessment

Quality assessment for all studies was conducted using the checklist provided in the National Institute for Health and Care Excellence (NICE) single technology appraisal template [60].

Table 4: Risk of Bias Assessment for First-line Studies

Study	Randomization	Concealment of treatment allocation	Blinding of care providers, participants, and outcome assessors	Groups similar at study outset	Unexpected imbalances in drop-out rates between groups	Selective reporting	ITT analysis included
Bonneterre 2004	+	−	−	+	+	+	+
Cinieri 2014	+	−	−	+	+	+	+
Finn 2015*	+	−	−	+	−	+	+
Finn 2016	?	+	?	+	+	+	+
Miles 2010	+	+	?	+	+	+	+
Mouridsen 2001	+	+	+	+	+	+	+
Nabholtz 2000	+	+	?	+	+	+	+
O'Shaughnessy 2001	+	−	−	+	+	+	+
Paridaens 2008	+	−	−	+	+	+	+
Robert 2011a	+	−	−	+	+	+	+
Robert 2011b	+	+	+	+	+	+	+
Robertson 2012*	+	−	−	+	+	+	+
Vici 2011	+	?	?	+	+	+	+
Welt 2016	+	−	−	+	+	+	+
Yardley 2009	?	?	?	+	+	+	+
Yardley 2015	?	?	?	+	+	+	+
**Forced connections**							
Ackland 2001	+	?	?	+	+	+	+
Bachelot 2011	?	?	?	−	+	+	+
Dixon 1992	?	?	?	+	+	+	+
Fountzilas 2009	+	?	?	−	+	−	+
Ghosn 2011	?	−	−	+	+	+	+
Heidemann 2002	+	?	?	+	+	+	+
Kaufmann 2000	+	+	?	+	+	+	+
Miller 2007	+	−	−	+	+	+	+

*Included in sensitivity analyses only.

+ = low risk of bias; − = high risk of bias; ? = unclear risk of bias; ITT = intention to treat.

Table 5: Risk of Bias Assessment for Second-line Studies

Study	Randomization	Concealment of treatment allocation	Blinding of care providers, participants, and outcome assessors	Groups similar at study outset	Unexpected imbalances in drop-out rates between groups	Selective reporting	ITT analysis included
Bachelot 2011	?	?	?	−	+	+	+
Baselga 2012	+	+	?	+	+	+	+
Bergh 2012	+	−	−	+	+	+	+
Brufsky 2011	+	−	−	+	+	+	+
Buzdar 1997	+	+	?	+	+	+	+
Buzdar 2001	−	+	+	+	+	+	+
Campone 2013	+	−	−	+	+	+	+
Chan 2009	+	?	?	+	+	+	+
Chia 2008	+	+	?	+	+	+	+
Cristofanilli 2016	+	+	+	+	+	+	+
Crown 2013	+	−	−	+	−	+	+
Del Mastro 2013	+	−	−	+	+	+	+
Di Leo 2010	+	+	?	+	+	+	+
Dombernowsky 1998	+	+	+	+	+	+	+
Fountzilas 2004	+	?	?	+	+	+	+
Fountzilas 2009	+	?	?	−	+	−	+
Gershanovich 1998	?	−	−	+	+	+	+
Ghosn 2011	?	−	−	+	+	+	+
Hatschek 2012	+	−	−	+	+	+	+
Heidemann 2002	+	?	?	+	+	+	+
Howell 2002	?	−	−	+	+	+	+
Jiang 2014	+	+	?	+	+	+	?
Jonat 1996	+	?	?	+	+	+	+
Jones 2005	+	−	−	+	+	+	+
Kaufmann 2000	+	+	?	+	+	+	+
Luck 2013	+	−	−	+	+	+	+
Martin 2011	+	+	+	+	+	−	+
Martin 2015	?	?	?	+	+	+	+
Miller 2007	+	−	−	+	+	+	+
Ohno 2010	?	+	?	+	+	+	+
Osborne 2002	?	+	?	+	+	+	+
Papadimitriou 2009	?	?	?	+	+	+	−
Pritchard 2010	?	+	?	+	+	+	+
Rugo 2013	+	−	−	+	+	−	+
Rugo 2015	+	?	?	+	+	+	+
Smorenburg 2014	+	?	?	+	+	+	+
Stockler 2011	+	?	?	+	+	+	+
Wang 2015	+	−	−	+	+	+	+
Xu 2011	?	+	?	+	+	+	+
Yardley 2013	+	+	?	+	−	+	+
**Forced connections**							
Ackland 2001	+	?	?	+	+	+	+
Bonneterre 2004	+	−	−	+	+	+	+

Study	Randomization	Concealment of treatment allocation	Blinding of care providers, participants, and outcome assessors	Groups similar at study outset	Unexpected imbalances in drop-out rates between groups	Selective reporting	ITT analysis included
Dixon 1992	?	?	?	+	+	+	+
Paridaens 2000	+	?	?	+	+	+	+

+ = low risk of bias; − = high risk of bias; ? = unclear risk of bias; ITT = intention to treat.

Appendix E: Sensitivity Analyses

Two key sensitivity analyses were conducted:

1) Fixed- and random-effects models of first-line PFS/TTP analysis using both PALOMA-1 and PALOMA-2
2) Fixed- and random-effects models excluding studies with the largest differences in median PFS/TTP for primary analyses.

Findings from these sensitivity analyses compared with the main analyses for first- and second-line PFS/TTP are summarized in **Table 6** and **Table 7**, respectively. Findings are sorted by highest SUCRA, based on the primary NMA, and by single agent versus combination therapies. Endocrine therapies are included in all analyses.

Table 6: Summary of Findings from Main Analyses and Sensitivity Analyses for First-line PFS/TTP: Palbociclib + Letrozole vs Comparators

Comparisons	Fixed-Effects Model	Random-Effects Model Using Vague Priors	Random-Effects Model Using Informative Priors	SA #1: PALOMA-1 and PALOMA-2 (FE)	SA #2: Largest Differences in Median Values Removed (FE)
Palbociclib + Letrozole	1	1	1	1	1
**Single Chemotherapy Agents and Endocrine Therapies**					
Anastrozole	**0.58 (0.41 to 0.83)**	0.55 (0.06 to 4.87)	0.57 (0.07 to 4.65)	**0.57 (0.41 to 0.79)**	**0.58 (0.41 to 0.83)**
Letrozole	**0.58 (0.46 to 0.72)**	0.58 (0.05 to 6.34)	0.55 (0.08 to 3.95)	**0.56 (0.46 to 0.68)**	**0.58 (0.46 to 0.72)**
Paclitaxel	0.59 (0.19 to 1.96)	0.59 (0.07 to 4.83)	0.63 (0.07 to 5.48)	0.57 (0.19 to 1.77)	0.59 (0.19 to 1.96)
Docetaxel	0.51 (0.14 to 2.03)	0.5 (0.06 to 3.92)	0.55 (0.07 to 4.24)	0.49 (0.16 to 1.55)	0.51 (0.14 to 2.03)
Exemestane	**0.47 (0.34 to 0.68)**	0.46 (0.05 to 3.96)	0.47 (0.07 to 3.38)	**0.46 (0.33 to 0.65)**	**0.47 (0.34 to 0.68)**
Tamoxifen	**0.4 (0.31 to 0.53)**	0.37 (0.04 to 3.15)	0.39 (0.05 to 3.17)	**0.39 (0.3 to 0.5)**	**0.4 (0.31 to 0.53)**
Fulvestrant 500 mg	NA	NA	NA	NA	NA
Megestrol Acetate	**0.38 (0.26 to 0.56)**	0.39 (0.05 to 3.13)	0.38 (0.04 to 3.76)	**0.37 (0.26 to 0.54)**	**0.38 (0.26 to 0.56)**
Capecitabine (Intermittent)	**0.28 (0.11 to 0.72)**	0.27 (0.03 to 2.12)	0.29 (0.03 to 2.45)	**0.28 (0.11 to 0.64)**	**0.28 (0.11 to 0.72)**
Mitoxantrone	**0.28 (0.13 to 0.61)**	0.27 (0.03 to 2.23)	0.28 (0.03 to 2.28)	**0.28 (0.13 to 0.54)**	**0.28 (0.13 to 0.61)**
**Combination Chemotherapy Agents and Endocrine Therapies**					
Paclitaxel + Bevacizumab + Everolimus	0.99 (0.29 to 3.81)	0.98 (0.12 to 8.43)	0.93 (0.12 to 7.17)	0.98 (0.29 to 3.44)	0.99 (0.29 to 3.81)
Paclitaxel + Bevacizumab	0.98 (0.31 to 3.38)	0.96 (0.13 to 7.29)	0.94 (0.1 to 8.65)	0.95 (0.31 to 3.06)	0.98 (0.31 to 3.38)
Docetaxel + Bevacizumab 15 mg	0.65 (0.18 to 2.69)	0.65 (0.09 to 4.7)	0.72 (0.09 to 6.11)	0.65 (0.18 to 2.35)	0.65 (0.18 to 2.69)
Docetaxel + Bevacizumab 7.5 mg	0.59 (0.16 to 2.4)	0.58 (0.08 to 4.19)	0.64 (0.08 to 5.36)	0.58 (0.16 to 2.11)	0.59 (0.16 to 2.4)
Paclitaxel + Sunitinib	0.6 (0.18 to 2.14)	0.6 (0.07 to 4.93)	0.66 (0.09 to 5)	0.58 (0.18 to 1.97)	0.6 (0.18 to 2.14)
Docetaxel + Gemcitabine	0.59 (0.2 to 1.91)	0.59 (0.08 to 4.32)	0.64 (0.08 to 5.2)	0.58 (0.2 to 1.73)	0.59 (0.2 to 1.91)
Liposomal Doxorubicin	0.54 (0.14 to 2.29)	0.53 (0.07 to 3.79)	0.59 (0.07 to 4.99)	0.53 (0.14 to 2.05)	0.54 (0.14 to 2.29)
Paclitaxel + Gemcitabine	0.51 (0.15 to 1.87)	0.49 (0.06 to 4.21)	0.56 (0.08 to 4.06)	0.5 (0.15 to 1.69)	0.51 (0.15 to 1.87)
Paclitaxel + Carboplatin	0.53 (0.17 to 1.83)	0.51 (0.06 to 4.35)	0.57 (0.06 to 5.16)	0.52 (0.17 to 1.68)	0.53 (0.17 to 1.83)
Docetaxel + Capecitabine	0.51 (0.19 to 1.49)	0.5 (0.06 to 4.19)	0.54 (0.08 to 3.9)	0.48 (0.19 to 1.15)	0.51 (0.19 to 1.49)
Capecitabine + Vinorelbine	0.5 (0.16 to 1.72)	0.49 (0.06 to 4.18)	0.55 (0.06 to 4.98)	0.5 (0.19 to 1.35)	0.5 (0.16 to 1.72)

Capecitabine + Bevacizumab + Vinorelbine	0.48 (0.19 to 1.27)	0.46 (0.06 to 3.76)	0.5 (0.06 to 4.02)	0.5 (0.14 to 1.79)	0.48 (0.19 to 1.27)
Docetaxel + Epirubicin	0.47 (0.2 to 1.19)	0.46 (0.06 to 3.32)	0.49 (0.06 to 4.15)	0.48 (0.2 to 1.06)	0.47 (0.2 to 1.19)
Capecitabine + Bevacizumab	0.4 (0.16 to 1.06)	0.39 (0.05 to 2.82)	0.41 (0.06 to 2.99)	**0.4 (0.16 to 0.96)**	0.4 (0.16 to 1.06)
Fluorouracil + Epirubicin + Cyclophosphamide	**0.33 (0.15 to 0.77)**	0.32 (0.04 to 2.47)	0.34 (0.03 to 3.34)	**0.34 (0.15 to 0.68)**	**0.33 (0.15 to 0.77)**
Cyclophosphamide + Methotrexate + 5-Fluorouracil	**0.24 (0.11 to 0.57)**	0.24 (0.03 to 2.11)	0.25 (0.03 to 1.85)	**0.25 (0.11 to 0.5)**	**0.24 (0.11 to 0.57)**
**Model Fit Statistics**	Residual Deviance = 25.08 vs 25<br>DIC = -0.02	Residual Deviance = 25.71 vs 25<br>DIC = 0.11<br>Heterogeneity (SD) = 0.67 (0.010 to 4.526)	Residual Deviance = 25.69 vs 25<br>DIC = -0.05<br>Heterogeneity (SD) = 0.73 (0.02 to 3.64)	Residual Deviance = 25.08 vs 26<br>DIC = -0.02	Residual Deviance = 25.08 vs 25<br>DIC = -0.02

Primary NMA uses data from PALOMA-2 [4]. Values shown are hazard ratios with 95% CrI. Statistically significant differences are shown in bold.

CrI = credible interval; DIC = deviance information criterion; FE = fixed-effects; NA = not applicable; SA = sensitivity analysis; SD = standard deviation.

Table 7: Summary of Findings from Main Analyses and Sensitivity Analyses for Second-line PFS/TTP: Palbociclib + Fulvestrant vs Comparators

Comparisons	Fixed-effects Model	Random-Effects Model Using Vague Priors	Random-Effects Model Using Informative Priors	SA #1: PALOMA-1 and PALOMA-2 (FE)	SA #2: Largest Differences in Median Values Removed (FE)
Palbociclib + Fulvestrant	1	1	1	NA	1
**Single Chemotherapy Agents and Endocrine Therapies**					
Doxorubicin	0.8 (0.27 to 2.44)	0.81 (0.22 to 3.02)	0.82 (0.19 to 3.4)	NA	0.92 (0.28 to 3.01)
Docetaxel	0.71 (0.24 to 2.13)	0.69 (0.2 to 2.57)	0.7 (0.17 to 2.76)	NA	0.83 (0.25 to 2.71)
Paclitaxel	0.48 (0.16 to 1.44)	0.49 (0.14 to 1.74)	0.49 (0.12 to 1.98)	NA	0.55 (0.17 to 1.77)
Fulvestrant 500 mg	**0.46 (0.36 to 0.59)**	**0.46 (0.32 to 0.65)**	**0.46 (0.32 to 0.67)**	NA	**0.46 (0.36 to 0.59)**
Fulvestrant 500/250 mg	**0.44 (0.31 to 0.65)**	**0.45 (0.27 to 0.75)**	**0.45 (0.28 to 0.77)**	NA	**0.46 (0.3 to 0.7)**
Exemestane	**0.43 (0.3 to 0.62)**	**0.44 (0.26 to 0.75)**	**0.44 (0.26 to 0.77)**	NA	**0.45 (0.3 to 0.67)**
Letrozole	**0.38 (0.26 to 0.56)**	**0.39 (0.23 to 0.7)**	**0.39 (0.23 to 0.72)**	NA	**0.37 (0.23 to 0.58)**
Anastrozole	**0.37 (0.27 to 0.51)**	**0.38 (0.25 to 0.61)**	**0.38 (0.24 to 0.62)**	NA	**0.39 (0.28 to 0.55)**
Fulvestrant 250 mg	**0.37 (0.28 to 0.49)**	**0.37 (0.25 to 0.57)**	**0.38 (0.25 to 0.58)**	NA	**0.37 (0.28 to 0.5)**
Megestrol Acetate	**0.34 (0.24 to 0.49)**	**0.35 (0.22 to 0.59)**	**0.35 (0.21 to 0.61)**	NA	**0.36 (0.24 to 0.54)**
Mitoxantrone	**0.26 (0.12 to 0.53)**	**0.26 (0.11 to 0.6)**	**0.26 (0.11 to 0.65)**	NA	**0.27 (0.12 to 0.58)**
Capecitabine (Continuous)	**0.24 (0.11 to 0.56)**	**0.25 (0.09 to 0.7)**	**0.25 (0.09 to 0.78)**	NA	**0.27 (0.11 to 0.66)**
Pegylated Liposomal Doxorubicin	**0.19 (0.07 to 0.5)**	**0.2 (0.06 to 0.64)**	**0.19 (0.06 to 0.67)**	NA	**0.19 (0.07 to 0.53)**
**Combination Chemotherapy Agents and Endocrine Therapies**					
Everolimus + Exemestane	1.13 (0.74 to 1.73)	1.15 (0.63 to 2.17)	1.16 (0.62 to 2.25)	NA	1.18 (0.74 to 1.88)
Paclitaxel + Bevacizumab + Gemcitabine	0.89 (0.28 to 2.82)	0.89 (0.23 to 3.49)	0.91 (0.2 to 3.95)	NA	0.86 (0.24 to 3.08)
Docetaxel + Sunitinib	0.77 (0.26 to 2.36)	0.75 (0.21 to 2.81)	0.76 (0.18 to 3.12)	NA	0.9 (0.27 to 3.01)
Paclitaxel + Bevacizumab	0.72 (0.24 to 2.2)	0.73 (0.2 to 2.68)	0.74 (0.17 to 3.11)	NA	0.7 (0.2 to 2.41)
Paclitaxel + Gemcitabine	0.64 (0.22 to 1.94)	0.67 (0.19 to 2.45)	0.67 (0.16 to 2.77)	NA	0.86 (0.24 to 3.08)
Ixabepilone 40 mg + Bevacizumab	0.61 (0.18 to 2.05)	0.62 (0.15 to 2.53)	0.62 (0.13 to 2.84)	NA	NA
Nab-Paclitaxel + Bevacizumab	0.6 (0.2 to 1.87)	0.6 (0.16 to 2.27)	0.61 (0.13 to 2.61)	NA	0.58 (0.17 to 2.04)
Docetaxel + Gemcitabine	0.55 (0.19 to 1.61)	0.57 (0.17 to 1.99)	0.58 (0.15 to 2.29)	NA	0.61 (0.19 to 1.93)

Paclitaxel + Motesanib	0.51 (0.16 to 1.61)	0.53 (0.14 to 2.13)	0.53 (0.12 to 2.33)	NA	0.58 (0.17 to 1.98)
Docetaxel + Capecitabine	0.49 (0.17 to 1.38)	0.51 (0.16 to 1.7)	0.51 (0.14 to 1.96)	NA	0.51 (0.17 to 1.58)
Docetaxel + Epirubicin	0.45 (0.19 to 1.06)	0.46 (0.17 to 1.28)	0.47 (0.16 to 1.44)	NA	0.47 (0.19 to 1.17)
Paclitaxel + Carboplatin	0.44 (0.14 to 1.38)	0.46 (0.13 to 1.67)	0.46 (0.11 to 1.94)	NA	0.5 (0.15 to 1.67)
Ixabepilone 16 mg + Bevacizumab	0.45 (0.15 to 1.39)	0.45 (0.12 to 1.71)	0.46 (0.1 to 1.93)	NA	NA
Capecitabine + Sorafenib	0.44 (0.17 to 1.14)	0.45 (0.14 to 1.49)	0.44 (0.14 to 1.54)	NA	0.43 (0.16 to 1.18)
Capecitabine + Vinorelbine	0.44 (0.15 to 1.28)	0.47 (0.14 to 1.62)	0.47 (0.13 to 1.85)	NA	0.72 (0.22 to 2.37)
Paclitaxel + Epirubicin	0.35 (0.11 to 1.16)	0.36 (0.09 to 1.44)	0.31 (0.07 to 1.37)	NA	0.4 (0.11 to 1.4)
Fluorouracil + Epirubicin + Cyclophosphamide	**0.31 (0.14 to 0.67)**	**0.32 (0.13 to 0.78)**	**0.31 (0.12 to 0.87)**	NA	**0.32 (0.14 to 0.73)**
Capecitabine (Intermittent)	**0.28 (0.13 to 0.65)**	**0.29 (0.1 to 0.81)**	**0.29 (0.1 to 0.89)**	NA	**0.28 (0.11 to 0.68)**
Paclitaxel + Capecitabine	**0.3 (0.09 to 0.98)**	0.31 (0.08 to 1.23)	0.31 (0.07 to 1.44)	NA	0.34 (0.09 to 1.2)
Paclitaxel + Epirubicin + Capecitabine	**0.29 (0.09 to 0.95)**	0.31 (0.08 to 1.16)	0.37 (0.08 to 1.7)	NA	0.33 (0.1 to 1.15)
Aminoglutethimide + Corticosteroid	**0.27 (0.17 to 0.43)**	**0.28 (0.15 to 0.55)**	**0.28 (0.15 to 0.57)**	NA	**0.26 (0.16 to 0.44)**
Capecitabine + Sunitinib	**0.23 (0.1 to 0.56)**	**0.24 (0.08 to 0.71)**	**0.23 (0.08 to 0.78)**	NA	**0.23 (0.09 to 0.58)**
Cyclophosphamide + Methotrexate + 5-Fluorouracil	**0.23 (0.1 to 0.51)**	**0.23 (0.09 to 0.61)**	**0.23 (0.09 to 0.68)**	NA	**0.24 (0.1 to 0.55)**
**Model Fit Statistics**	Residual Deviance = 58.12 vs 51<br>DIC = -4.11	Residual Deviance = 52.50 vs 51<br>DIC = -4.36<br>Heterogeneity (SD) = 0.11 (0.01 to 0.26)	Residual Deviance = 52.11 vs 51<br>DIC = -4.63<br>Heterogeneity (SD) = 0.11 (0.01 to 0.26)	NA	Residual Deviance = 39.47 vs 39<br>DIC = -3.12

Primary NMA uses data from PALOMA-3 [33]. Values shown are hazard ratios with 95% CrI. Statistically significant differences are shown in bold.

CrI = credible interval; DIC = deviance information criterion; FE = fixed-effects; NA = not applicable; SA = sensitivity analysis; SD = standard deviation.

1) ***Fixed- and random-effects models of first-line PFS/TTP analysis using both PALOMA-1 and PALOMA-2***

In the first-line fixed-effects model using both PALOMA-1 and PALOMA-2, palbociclib + letrozole was associated with improvements in PFS/TTP relative to all other treatments (**Table 8**). Palbociclib + letrozole showed statistically significant improvements in PFS/TTP relative to capecitabine (intermittent: HR = 0.28 [0.11 to 0.64]) and mitoxantrone (HR = 0.28 [0.13 to 0.54]), and trended toward improvements (not statistically significant) versus paclitaxel (HR = 0.57 [0.19 to 1.77]), docetaxel (HR = 0.5 [0.14 to 1.79]) and other monotherapy or combination chemotherapy agents (HRs ranging from 0.25 to 0.98). Palbociclib + letrozole was associated with the highest SUCRA value among all treatments (96.0%) and a mean rank value closest to 1. The probability of treatment being best was 42.8% for palbociclib + letrozole; all remaining treatments were associated with values below 30.0%.

Table 8: First-line Fixed-effects NMA Results for PFS/TTP: Palbociclib + Letrozole vs Comparators – Both PALOMA-1 and PALOMA-2

Comparisons	HR (95% CrI) Fixed-effects Model	SUCRA	Probability Best	Mean Rank (95% CrI)
Palbociclib + Letrozole	1	96.00%	42.84%	2 (1 to 14)
**Single Chemotherapy Agents and Endocrine Therapies**				
**Anastrozole**	**0.57 (0.41 to 0.79)**	**64.00%**	**0.01%**	**10 (2 to 21)**
**Letrozole**	**0.56 (0.46 to 0.68)**	**64.00%**	**0.00%**	**10 (2 to 21)**
Paclitaxel	0.57 (0.19 to 1.77)	64.00%	0.00%	10 (4 to 22)
Docetaxel	0.5 (0.14 to 1.79)	48.00%	0.00%	14 (4 to 26)
**Exemestane**	**0.46 (0.33 to 0.65)**	**44.00%**	**0.00%**	**15 (4 to 23)**
**Tamoxifen**	**0.39 (0.3 to 0.5)**	**28.00%**	**0.00%**	**19 (6 to 26)**
**Megestrol Acetate**	**0.37 (0.26 to 0.54)**	**24.00%**	**0.00%**	**20 (7 to 26)**
**Capecitabine (Intermittent)**	**0.28 (0.11 to 0.64)**	**8.00%**	**0.00%**	**24 (12 to 26)**
**Mitoxantrone**	**0.28 (0.13 to 0.54)**	**8.00%**	**0.00%**	**24 (14 to 26)**
**Combination Chemotherapy Agents and Endocrine Therapies**				
Paclitaxel + Bevacizumab + Everolimus	0.98 (0.29 to 3.44)	96.00%	29.70%	2 (1 to 14)
Paclitaxel + Bevacizumab	0.95 (0.31 to 3.06)	92.00%	16.88%	3 (1 to 11)
Docetaxel + Bevacizumab 15 mg	0.65 (0.18 to 2.35)	76.00%	5.80%	7 (1 to 22)
Docetaxel + Bevacizumab 7.5 mg	0.58 (0.16 to 2.11)	64.00%	1.10%	10 (2 to 24)
Paclitaxel + Sunitinib	0.58 (0.18 to 1.97)	64.00%	0.04%	10 (3 to 24)
Docetaxel + Gemcitabine	0.58 (0.2 to 1.73)	64.00%	0.03%	10 (4 to 20)
Liposomal Doxorubicin	0.53 (0.14 to 2.05)	56.00%	2.02%	12 (2 to 26)
Paclitaxel + Gemcitabine	0.5 (0.15 to 1.69)	52.00%	0.47%	13 (3 to 26)
Paclitaxel + Carboplatin	0.52 (0.17 to 1.68)	52.00%	0.04%	13 (4 to 25)
Capecitabine + Bevacizumab + Vinorelbine	0.48 (0.19 to 1.15)	48.00%	0.91%	14 (2 to 22)
Docetaxel + Capecitabine	0.5 (0.19 to 1.35)	48.00%	0.05%	14 (4 to 23)

Capecitabine + Vinorelbine	0.49 (0.16 to 1.55)	48.00%	0.02%	14 (5 to 25)
Docetaxel + Epirubicin	0.48 (0.2 to 1.06)	44.00%	0.08%	15 (4 to 22)
**Capecitabine + Bevacizumab**	**0.4 (0.16 to 0.96)**	**32.00%**	**0.01%**	**18 (4 to 24)**
**Fluorouracil + Epirubicin + Cyclophosphamide**	**0.34 (0.15 to 0.68)**	**20.00%**	**0.00%**	**21 (10 to 24)**
**Cyclophosphamide + Methotrexate + 5-Fluorouracil**	**0.25 (0.11 to 0.5)**	**4.00%**	**0.00%**	**25 (18 to 26)**
**Model Fit Statistics**	Residual deviance = 25.42 vs 26<br>DIC = -1.04			

Statistically significant differences are shown in bold.
CrI = credible interval; DIC = deviance information criterion; HR = hazard ratio; SUCRA = Surface Under the Cumulative RAnking curve.

Palbociclib + letrozole was also associated with improvements in PFS/TTP relative to all other treatments in the first-line random-effects model using vague priors including both PALOMA-1 and PALOMA-2 (**Table 9**). Although statistical significance was not observed, palbociclib + letrozole was associated with the highest SUCRA value among all treatments (84.0%), and the highest probability of being the best treatment (23.5%).

Table 9: First-line Random-effects (Vague Priors) NMA Results for PFS/TTP: Palbociclib + Letrozole vs Comparators – Both PALOMA-1 and PALOMA-2

Comparisons	HR (95% CrI) Random-effects Model: Vague Priors	SUCRA	Probability Best	Mean Rank (95% CrI)
Palbociclib + Letrozole	1	84.00%	23.45%	5 (1 to 25)
**Single Chemotherapy Agents and Endocrine Therapies**				
Paclitaxel	0.57 (0.07 to 4.83)	60.00%	0.67%	11 (3 to 25)
Anastrozole	0.55 (0.06 to 4.87)	56.00%	5.49%	12 (1 to 26)
Letrozole	0.55 (0.05 to 6.34)	56.00%	1.77%	12 (2 to 26)
Liposomal Doxorubicin	0.53 (0.07 to 3.79)	52.00%	5.04%	13 (1 to 26)
Docetaxel	0.5 (0.06 to 3.92)	52.00%	0.55%	13 (3 to 25)
Exemestane	0.46 (0.05 to 3.96)	44.00%	1.37%	15 (2 to 25)
Megestrol Acetate	0.37 (0.04 to 3.15)	36.00%	0.95%	17 (3 to 26)
Tamoxifen	0.39 (0.05 to 3.13)	36.00%	0.63%	17 (3 to 26)
Capecitabine (Intermittent)	0.27 (0.03 to 2.12)	24.00%	0.90%	20 (3 to 26)
Mitoxantrone	0.27 (0.03 to 2.23)	24.00%	0.51%	20 (4 to 26)
**Combination Chemotherapy Agents and Endocrine Therapies**				
Paclitaxel + Bevacizumab + Everolimus	0.98 (0.12 to 8.43)	80.00%	18.15%	6 (1 to 26)
Paclitaxel + Bevacizumab	0.96 (0.13 to 7.29)	80.00%	9.28%	6 (1 to 24)
Docetaxel + Bevacizumab 15 mg	0.65 (0.09 to 4.7)	64.00%	6.92%	10 (1 to 26)
Docetaxel + Bevacizumab 7.5 mg	0.58 (0.08 to 4.19)	60.00%	4.56%	11 (1 to 26)
Paclitaxel + Sunitinib	0.6 (0.07 to 4.93)	60.00%	3.83%	11 (1 to 26)
Docetaxel + Gemcitabine	0.57 (0.08 to 4.32)	60.00%	0.34%	11 (3 to 23)
Paclitaxel + Gemcitabine	0.49 (0.06 to 4.21)	52.00%	2.12%	13 (2 to 26)
Paclitaxel + Carboplatin	0.51 (0.06 to 4.35)	52.00%	1.55%	13 (2 to 26)

Docetaxel + Capecitabine	0.5 (0.06 to 4.19)	52.00%	0.80%	13 (3 to 24)
Capecitabine + Vinorelbine	0.49 (0.06 to 4.18)	52.00%	0.58%	13 (3 to 25)
Capecitabine + Bevacizumab + Vinorelbine	0.46 (0.06 to 3.76)	48.00%	6.67%	14 (1 to 26)
Docetaxel + Epirubicin	0.46 (0.06 to 3.32)	48.00%	0.80%	14 (3 to 24)
Capecitabine + Bevacizumab	0.39 (0.05 to 2.82)	40.00%	2.26%	16 (2 to 26)
Fluorouracil + Epirubicin + Cyclophosphamide	0.32 (0.04 to 2.47)	32.00%	0.29%	18 (4 to 25)
Cyclophosphamide + Methotrexate + 5-Fluorouracil	0.24 (0.03 to 2.11)	20.00%	0.51%	21 (4 to 26)
**Model Fit Statistics**	Residual Deviance = 25.71 vs 26<br>DIC = 0.11<br>Heterogeneity (SD) = 0.67 (0.01 to 4.53)			

CrI = credible interval; DIC = deviance information criterion; HR = hazard ratio; SD = standard deviation; SUCRA = Surface Under the Cumulative RAnking curve.

2) Fixed- and random-effects models excluding studies with the largest differences in median PFS/TTP for primary first- and second-line analyses

Some of the connecting nodes in the treatment network (i.e., those with connections to multiple interventions in the network and multiple studies which can be compared) were noted to have variable median PFS/TTP durations, despite the administration of equivalent interventions. This is likely a result of differences in patient characteristics. As a sensitivity analysis, we excluded studies wherein the largest differences in median PFS/TTP were observed. For example, if there were two studies in one connection and they went into a node, we kept the study that was most similar in terms of median PFS/TTP to other connections leaving the same node.

This sensitivity analysis was not possible for the first-line PFS/TTP network, since it is only comprised of single study connections. A comparison of observed median values for second-line PFS/TTP is shown in **Figure 1**, with excluded studies circled. Rugo 2013, Miller 2007, Wang 2015, Martin 2015, Dombernowsky 1998, Buzdar 1997, Osborne 2002, Jiang 2014, and Ohno 2010 were excluded due to the largest differences in median PFS/TTP values. The second-line fixed-effects NMA results suggest that palbociclib + fulvestrant is associated with improvements in PFS/TTP relative to all other treatments (**Table 10**). Palbociclib + fulvestrant showed statistically significant improvements in PFS/TTP relative to capecitabine (continuous: HR = 0.27 [0.11 to 0.66]; intermittent: HR = 0.28 [0.11 to 0.68]) and mitoxantrone (HR = 0.27 [0.12 to 0.58]), and trended toward improvements (not statistically significant) versus docetaxel (HR = 0.83 [0.25 to 2.71]), paclitaxel (HR = 0.55 [0.17 to 1.77]), and other monotherapy or combination chemotherapy agents (HRs ranging from 0.19 to 1.18). With the exception of everolimus + exemestane, palbociclib + fulvestrant was associated with the highest SUCRA value among all treatments (94.1%), and a probability best of 14.6%.

Figure 1: Comparison of Observed Median Values for Second-line PFS/TTP by Treatment and Study

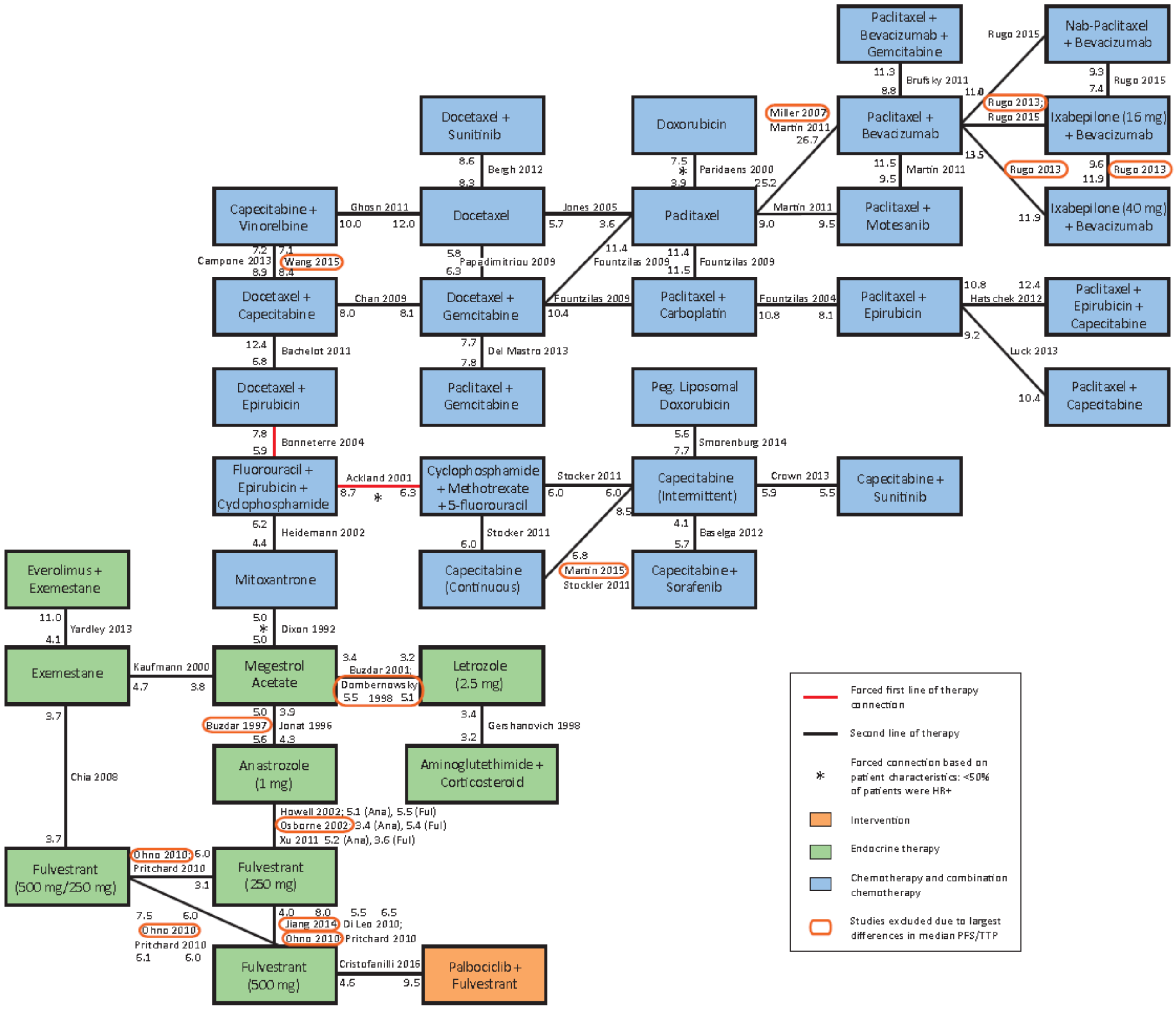

Median PFS/TTP values (months) are labelled.

HR+ = hormone receptor-positive; PFS = progression-free survival; TTP = time to progression.

Table 10: Second-line Fixed-effects NMA Results for PFS/TTP: Palbociclib + Fulvestrant vs Comparators – Studies with Largest Median Survival Time Differences Removed

Comparisons	HR (95% CrI) Fixed-effects Model	SUCRA	Probability Best	Mean Rank (95% CrI)
Palbociclib + Fulvestrant	1	94.12%	14.64%	3 (1 to 18)
**Single Chemotherapy Agents and Endocrine Therapies**				
Doxorubicin	0.92 (0.28 to 3.01)	91.18%	11.05%	4 (1 to 13)
Docetaxel	0.83 (0.25 to 2.71)	88.24%	0.91%	5 (2 to 15)
Paclitaxel	0.55 (0.17 to 1.77)	61.76%	0.00%	14 (9 to 27)
**Fulvestrant 500/250 mg**	**0.46 (0.3 to 0.7)**	**52.94%**	**0.00%**	**17 (3 to 28)**
**Exemestane**	**0.45 (0.3 to 0.67)**	**50.00%**	**0.00%**	**18 (4 to 28)**
**Fulvestrant 500 mg**	**0.46 (0.36 to 0.59)**	**50.00%**	**0.00%**	**18 (3 to 29)**
**Anastrozole**	**0.39 (0.28 to 0.55)**	**38.24%**	**0.00%**	**22 (6 to 32)**
**Fulvestrant 250 mg**	**0.37 (0.28 to 0.5)**	**32.35%**	**0.00%**	**24 (7 to 34)**
**Letrozole**	**0.37 (0.23 to 0.58)**	**32.35%**	**0.00%**	**24 (7 to 33)**
**Megestrol Acetate**	**0.36 (0.24 to 0.54)**	**32.35%**	**0.00%**	**24 (8 to 33)**
**Capecitabine (Continuous)**	**0.27 (0.11 to 0.66)**	**17.65%**	**0.00%**	**29 (18 to 34)**
**Capecitabine (Intermittent)**	**0.28 (0.11 to 0.68)**	**17.65%**	**0.00%**	**29 (17 to 33)**
**Mitoxantrone**	**0.27 (0.12 to 0.58)**	**14.71%**	**0.00%**	**30 (20 to 34)**
**Pegylated Liposomal Doxorubicin**	**0.19 (0.07 to 0.53)**	**0.00%**	**0.00%**	**35 (22 to 35)**
**Combination Chemotherapy Agents and Endocrine Therapies**				
Everolimus + Exemestane	1.18 (0.74 to 1.88)	97.06%	45.93%	2 (1 to 16)
Docetaxel + Sunitinib	0.9 (0.27 to 3.01)	91.18%	11.25%	4 (1 to 14)
Paclitaxel + Bevacizumab + Gemcitabine	0.86 (0.24 to 3.08)	88.24%	13.08%	5 (1 to 19)
Capecitabine + Vinorelbine	0.72 (0.22 to 2.37)	79.41%	1.67%	8 (2 to 21)
Paclitaxel + Gemcitabine	0.71 (0.22 to 2.32)	79.41%	0.80%	8 (3 to 21)
Paclitaxel + Bevacizumab	0.7 (0.2 to 2.41)	76.47%	0.22%	9 (3 to 23)
Docetaxel + Gemcitabine	0.61 (0.19 to 1.93)	70.59%	0.00%	11 (7 to 23)
Paclitaxel + Motesanib	0.58 (0.17 to 1.98)	64.71%	0.18%	13 (4 to 29)
Nab-Paclitaxel + Bevacizumab	0.58 (0.17 to 2.04)	64.71%	0.01%	13 (5 to 29)
Paclitaxel + Carboplatin	0.5 (0.15 to 1.67)	55.88%	0.00%	16 (8 to 31)
Docetaxel + Capecitabine	0.51 (0.17 to 1.58)	55.88%	0.00%	16 (10 to 28)
Docetaxel + Epirubicin	0.47 (0.19 to 1.17)	52.94%	0.01%	17 (7 to 27)
Capecitabine + Sorafenib	0.43 (0.16 to 1.18)	47.06%	0.24%	19 (3 to 31)
Paclitaxel + Epirubicin	0.4 (0.11 to 1.4)	41.18%	0.01%	21 (10 to 33)
**Fluorouracil + Epirubicin + Cyclophosphamide**	**0.32 (0.14 to 0.73)**	**29.41%**	**0.00%**	**25 (15 to 31)**
Paclitaxel + Capecitabine	0.34 (0.09 to 1.2)	26.47%	0.00%	26 (14 to 35)
Paclitaxel + Epirubicin + Capecitabine	0.33 (0.1 to 1.15)	26.47%	0.00%	26 (16 to 35)
**Aminoglutethimide + Corticosteroid**	**0.26 (0.16 to 0.44)**	**14.71%**	**0.00%**	**30 (13 to 35)**
**Cyclophosphamide + Methotrexate + 5-Fluorouracil**	**0.24 (0.1 to 0.55)**	**8.82%**	**0.00%**	**32 (24 to 35)**
**Capecitabine + Sunitinib**	**0.23 (0.09 to 0.58)**	**5.88%**	**0.00%**	**33 (21 to 35)**

**Model Fit Statistics**	Residual Deviance = 39.47 vs 39<br>DIC = -3.12

Statistically significant differences are shown in bold.

CrI = credible interval; DIC = deviance information criterion; HR = hazard ratio; SUCRA = Surface Under the Cumulative RAnking curve.

With the exception of everolimus + exemestane, palbociclib + fulvestrant was associated with improvements in PFS/TTP relative to all other treatments in the second-line random-effects model using vague priors, including fulvestrant (500 mg) (**Table 11**). With the exception of everolimus + exemestane, palbociclib + fulvestrant was associated with the highest SUCRA value among all treatments (94.1%), and a probability best of 18.4%.

Table 11: Second-line Random-effects (Vague Priors) NMA Results for PFS/TTP: Palbociclib + Fulvestrant vs Comparators – Studies with Largest Median Survival Time Differences Removed

Comparisons	HR (95% CrI) Random-effects Model: Vague Priors	SUCRA	Probability Best	Mean Rank (95% CrI)
Palbociclib + Fulvestrant	1	94.12%	18.42%	3 (1 to 20)
**Single Chemotherapy Agents and Endocrine Therapies**				
Doxorubicin	0.86 (0.2 to 4.21)	91.18%	10.71%	4 (1 to 19)
Docetaxel	0.77 (0.19 to 3.45)	85.29%	0.74%	6 (2 to 20)
Paclitaxel	0.52 (0.13 to 2.36)	58.82%	0.00%	15 (8 to 29)
**Fulvestrant 500/250 mg**	**0.46 (0.23 to 0.91)**	**52.94%**	**0.02%**	**17 (3 to 31)**
**Fulvestrant 500 mg**	**0.46 (0.29 to 0.71)**	**52.94%**	**0.01%**	**17 (3 to 32)**
**Exemestane**	**0.45 (0.22 to 0.91)**	**50.00%**	**0.00%**	**18 (4 to 30)**
**Anastrozole**	**0.4 (0.22 to 0.75)**	**41.18%**	**0.01%**	**21 (5 to 33)**
**Letrozole**	**0.37 (0.16 to 0.83)**	**35.29%**	**0.03%**	**23 (5 to 34)**
**Fulvestrant 250 mg**	**0.37 (0.22 to 0.64)**	**35.29%**	**0.00%**	**23 (6 to 34)**
**Megestrol Acetate**	**0.37 (0.18 to 0.74)**	**32.35%**	**0.00%**	**24 (8 to 33)**
Capecitabine (Intermittent)	0.28 (0.08 to 1.03)	20.59%	0.01%	28 (11 to 33)
**Capecitabine (Continuous)**	**0.27 (0.08 to 0.98)**	**17.65%**	**0.03%**	**29 (11 to 35)**
**Mitoxantrone**	**0.27 (0.1 to 0.76)**	**17.65%**	**0.00%**	**29 (15 to 35)**
**Pegylated Liposomal Doxorubicin**	**0.19 (0.05 to 0.79)**	**2.94%**	**0.02%**	**34 (16 to 35)**
**Combination Chemotherapy Agents and Endocrine Therapies**				
Everolimus + Exemestane	1.19 (0.52 to 2.72)	97.06%	41.23%	2 (1 to 18)
Paclitaxel + Bevacizumab + Gemcitabine	0.81 (0.17 to 4.21)	88.24%	12.16%	5 (1 to 26)
Docetaxel + Sunitinib	0.83 (0.19 to 4.02)	88.24%	8.98%	5 (1 to 22)
Capecitabine + Vinorelbine	0.69 (0.17 to 3.09)	79.41%	2.19%	8 (2 to 24)
Paclitaxel + Gemcitabine	0.68 (0.16 to 3.24)	76.47%	2.06%	9 (2 to 25)
Paclitaxel + Bevacizumab	0.67 (0.15 to 3.23)	76.47%	0.48%	9 (2 to 28)
Docetaxel + Gemcitabine	0.58 (0.15 to 2.65)	67.65%	0.02%	12 (5 to 25)
Nab-Paclitaxel + Bevacizumab	0.56 (0.12 to 2.83)	64.71%	0.50%	13 (3 to 32)

Paclitaxel + Motesanib	0.55 (0.12 to 2.74)	61.76%	0.45%	14 (3 to 31)
Paclitaxel + Carboplatin	0.49 (0.11 to 2.37)	55.88%	0.07%	16 (6 to 31)
Docetaxel + Capecitabine	0.49 (0.13 to 2.11)	55.88%	0.01%	16 (8 to 29)
Docetaxel + Epirubicin	0.46 (0.14 to 1.6)	50.00%	0.06%	18 (5 to 28)
Capecitabine + Sorafenib	0.44 (0.11 to 1.78)	47.06%	1.20%	19 (2 to 32)
Paclitaxel + Epirubicin + Capecitabine	0.39 (0.08 to 2.11)	38.24%	0.32%	22 (5 to 34)
Paclitaxel + Capecitabine	0.33 (0.07 to 1.81)	29.41%	0.15%	25 (8 to 35)
Fluorouracil + Epirubicin + Cyclophosphamide	0.32 (0.11 to 1.01)	29.41%	0.00%	25 (12 to 32)
Paclitaxel + Epirubicin + Capecitabine	0.32 (0.07 to 1.68)	26.47%	0.01%	26 (12 to 35)
**Aminoglutethimide + Corticosteroid**	**0.27 (0.11 to 0.67)**	**17.65%**	**0.05%**	**29 (10 to 35)**
**Cyclophosphamide + Methotrexate + 5-Fluorouracil**	**0.24 (0.07 to 0.79)**	**11.76%**	**0.01%**	**31 (18 to 35)**
**Capecitabine + Sunitinib**	**0.23 (0.06 to 0.91)**	**8.82%**	**0.04%**	**32 (13 to 35)**
**Model Fit Statistics**	Residual Deviance = 38.34 vs 39<br>DIC = -2.18<br>Heterogeneity (SD) = 0.14 (0.003 to 0.45)			

Statistically significant differences are shown in bold.

CrI = credible interval; DIC = deviance information criterion; HR = hazard ratio; SD = standard deviation; SUCRA = Surface Under the Cumulative RAnking curve.

Appendix F: PRISMA NMA Checklist

Table 12: PRISMA NMA Checklist of Items to Include When Reporting a Systematic Review Involving a Network Meta-analysis

Section/ Topic	Item #	Checklist Item	Reported on Page #
**TITLE**			
Title	1	Identify the report as a systematic review *incorporating a network meta-analysis (or related form of meta-analysis).*	1
**ABSTRACT**			
Structured summary	2	Provide a structured summary including, as applicable:<br>**Background:** main objectives<br>**Methods:** data sources; study eligibility criteria, participants, and interventions; study appraisal; and *synthesis methods, such as network meta-analysis.*<br>**Results:** number of studies and participants identified; summary estimates with corresponding confidence/credible intervals; *treatment rankings may also be discussed. Authors may choose to summarize pairwise comparisons against a chosen treatment included in their analyses for brevity.*<br>**Discussion/Conclusions:** limitations; conclusions and implications of findings.<br>**Other:** primary source of funding; systematic review registration number with registry name.	2
**INTRODUCTION**			
Rationale	3	Describe the rationale for the review in the context of what is already known*, including mention of why a network meta-analysis has been conducted.*	4
Objectives	4	Provide an explicit statement of questions being addressed, with reference to population, interventions, comparisons, outcomes, and study design (PICOS).	4
**METHODS**			
Protocol and registration	5	Indicate whether a review protocol exists and if and where it can be accessed (e.g., Web address); and, if available, provide registration information, including registration number.	NA
Eligibility criteria	6	Specify study characteristics (e.g., PICOS, length of follow-up) and report characteristics (e.g., years considered, language, publication status) used as criteria for eligibility, giving rationale. *Clearly describe eligible treatments included in the treatment network, and note whether any have been clustered or merged into the same node (with justification).*	4-5
Information sources	7	Describe all information sources (e.g., databases with dates of coverage, contact with study authors to identify additional studies) in the search and date last searched.	4

Search	8	Present full electronic search strategy for at least one database, including any limits used, such that it could be repeated.	4, Appendix A
Study selection	9	State the process for selecting studies (i.e., screening, eligibility, included in systematic review, and, if applicable, included in the meta-analysis).	5, Appendix B
Data collection process	10	Describe method of data extraction from reports (e.g., piloted forms, independently, in duplicate) and any processes for obtaining and confirming data from investigators.	5
Data items	11	List and define all variables for which data were sought (e.g., PICOS, funding sources) and any assumptions and simplifications made.	5, Appendix B
Geometry of the network	**S1**	Describe methods used to explore the geometry of the treatment network under study and potential biases related to it. This should include how the evidence base has been graphically summarized for presentation, and what characteristics were compiled and used to describe the evidence base to readers.	5
Risk of bias within individual studies	12	Describe methods used for assessing risk of bias of individual studies (including specification of whether this was done at the study or outcome level), and how this information is to be used in any data synthesis.	6, Appendix D
Summary measures	13	State the principal summary measures (e.g., risk ratio, difference in means). *Also describe the use of additional summary measures assessed, such as treatment rankings and surface under the cumulative ranking curve (SUCRA) values, as well as modified approaches used to present summary findings from meta-analyses.*	5-6
Planned methods of analysis	14	Describe the methods of handling data and combining results of studies for each network meta-analysis. This should include, but not be limited to:<br>• *Handling of multi-arm trials;*<br>• *Selection of variance structure;*<br>• *Selection of prior distributions in Bayesian analyses; and*<br>• *Assessment of model fit.*	5-6
Assessment of Inconsistency	**S2**	Describe the statistical methods used to evaluate the agreement of direct and indirect evidence in the treatment network(s) studied. Describe efforts taken to address its presence when found.	6
Risk of bias across studies	15	Specify any assessment of risk of bias that may affect the cumulative evidence (e.g., publication bias, selective reporting within studies).	6, Appendix D
Additional analyses	16	Describe methods of additional analyses if done, indicating which were pre-specified. This may include, but not be limited to, the following:<br>• Sensitivity or subgroup analyses;<br>• Meta-regression analyses;<br>• *Alternative formulations of the treatment network; and*<br>• *Use of alternative prior distributions for Bayesian analyses (if applicable).*	6, Appendix E

RESULTS**

Study selection	17	Give numbers of studies screened, assessed for eligibility, and included in the review, with reasons for exclusions at each stage, ideally with a flow diagram.	6-8, Figure 1
**Presentation of network structure**	**S3**	Provide a network graph of the included studies to enable visualization of the geometry of the treatment network.	Figures 2-3
**Summary of network geometry**	**S4**	Provide a brief overview of characteristics of the treatment network. This may include commentary on the abundance of trials and randomized patients for the different interventions and pairwise comparisons in the network, gaps of evidence in the treatment network, and potential biases reflected by the network structure.	6-8
Study characteristics	18	For each study, present characteristics for which data were extracted (e.g., study size, PICOS, follow-up period) and provide the citations.	7-8
Risk of bias within studies	19	Present data on risk of bias of each study and, if available, any outcome level assessment.	7, Appendix D
Results of individual studies	20	For all outcomes considered (benefits or harms), present, for each study: 1) simple summary data for each intervention group, and 2) effect estimates and confidence intervals. *Modified approaches may be needed to deal with information from larger networks.*	Available upon request
Synthesis of results	21	Present results of each meta-analysis done, including confidence/credible intervals. *In larger networks, authors may focus on comparisons versus a particular comparator (e.g., placebo or standard care), with full findings presented in an appendix. League tables and forest plots may be considered to summarize pairwise comparisons.* If additional summary measures were explored (such as treatment rankings), these should also be presented.	7-8
**Exploration for inconsistency**	**S5**	Describe results from investigations of inconsistency. This may include such information as measures of model fit to compare consistency and inconsistency models, *P* values from statistical tests, or summary of inconsistency estimates from different parts of the treatment network.	7-8
Risk of bias across studies	22	Present results of any assessment of risk of bias across studies for the evidence base being studied.	7, Appendix D
Results of additional analyses	23	Give results of additional analyses, if done (e.g., sensitivity or subgroup analyses, meta-regression analyses*, alternative network geometries studied, alternative choice of prior distributions for Bayesian analyses,* and so forth).	8, Appendix E
**DISCUSSION**			
Summary of evidence	24	Summarize the main findings, including the strength of evidence for each main outcome; consider their relevance to key groups (e.g., healthcare providers, users, and policy-makers).	8-10
Limitations	25	Discuss limitations at study and outcome level (e.g., risk of bias), and at review level (e.g., incomplete retrieval of identified research, reporting bias). *Comment on the validity of the assumptions, such as transitivity and consistency. Comment on any concerns regarding network geometry (e.g., avoidance of certain comparisons).*	9-10
Conclusions	26	Provide a general interpretation of the results in the context of other evidence, and implications for future research.	10

**FUNDING**			
Funding	27	Describe sources of funding for the systematic review and other support (e.g., supply of data); role of funders for the systematic review. This should also include information regarding whether funding has been received from manufacturers of treatments in the network and/or whether some of the authors are content experts with professional conflicts of interest that could affect use of treatments in the network.	1, 10

*Text in italics indicates wording specific to reporting of network meta-analyses that has been added to guidance from the PRISMA statement.

**Authors may wish to plan for use of appendices to present all relevant information in full detail for items in this section.

PICOS = population, intervention, comparators, outcomes, study design

REFERENCES

1. Bonneterre J, Dieras V, Tubiana-Hulin M, Bougnoux P, Bonneterre ME, Delozier T, Mayer F, Culine S, Dohoulou N, Bendahmane B (2004) Phase II multicentre randomised study of docetaxel plus epirubicin vs 5-fluorouracil plus epirubicin and cyclophosphamide in metastatic breast cancer. Br J Cancer 91 (8):1466-1471. doi:10.1038/sj.bjc.6602179

2. Cinieri S, Chan A, Altundag K, Vandebroek A, Tubiana-Mathieu N, Barnadas A, Dodyk P, Lazzarelli S, Botha M, Rauch D, Khasanov R, Slabber CF, Bougnoux P, Cardenas JS, Castellanos Diez J, Schmidt M, Godes MJ, Chen DR, Villanova G, Coskun U (2014) Three-arm randomised phase II study evaluating oral vinorelbine plus capecitabine versus paclitaxel plus gemcitabine versus docetaxel plus gemcitabine as first-line chemotherapy in patients with metastatic breast cancer: final results (NorCap-CA223 trial). ASCO 50th Annual Meeting

3. Finn RS, Crown JP, Lang I, Boer K, Bondarenko IM, Kulyk SO, Ettl J, Patel R, Pinter T, Schmidt M, Shparyk Y, Thummala AR, Voytko NL, Fowst C, Huang X, Kim ST, Randolph S, Slamon DJ (2015) The cyclin-dependent kinase 4/6 inhibitor palbociclib in combination with letrozole versus letrozole alone as first-line treatment of oestrogen receptor-positive, HER2-negative, advanced breast cancer (PALOMA-1/TRIO-18): a randomised phase 2 study. Lancet Oncol 16 (1):25-35. doi:10.1016/S1470-2045(14)71159-3

4. Finn RS, Martin M, Rugo H, Jones S, Im SA, Gelmon K, Harbeck N, Lipatov ON, Walshe JM, Moulder S, Gauthier E, Lu DR, Randolph S, Dieras V, Slamon DJ (2016) Palbociclib and letrozole in advanced breast cancer. The New England journal of medicine 375 (20):1925-1936

5. Miles DW, Chan A, Dirix LY, Cortes J, Pivot X, Tomczak P, Delozier T, Sohn JH, Provencher L, Puglisi F, Harbeck N, Steger GG, Schneeweiss A, Wardley AM, Chlistalla A, Romieu G (2010) Phase III study of bevacizumab plus docetaxel compared with placebo plus docetaxel for the first-line treatment of human epidermal growth factor receptor 2-negative metastatic breast cancer. J Clin Oncol 28 (20):3239-3247. doi:10.1200/JCO.2008.21.6457

6. Mouridsen H, Gershanovich M, Sun Y, Perez-Carrion R, Boni C, Monnier A, Apffelstaedt J, Smith R, Sleeboom HP, Janicke F, Pluzanska A, Dank M, Becquart D, Bapsy PP, Salminen E, Snyder R, Lassus M, Verbeek JA, Staffler B, Chaudri-Ross HA, Dugan M (2001) Superior efficacy of letrozole versus tamoxifen as first-line therapy for postmenopausal women with advanced breast cancer: results of a phase III study of the International Letrozole Breast Cancer Group. J Clin Oncol 19 (10):2596-2606

7. Nabholtz JM, Buzdar A, Pollak M, Harwin W, Burton G, Mangalik A, Steinberg M, Webster A, von Euler M (2000) Anastrozole is superior to tamoxifen as first-line therapy for advanced breast cancer in postmenopausal women: results of a North American multicenter randomized trial. Arimidex Study Group. J Clin Oncol 18 (22):3758-3767

8. O'Shaughnessy JA, Blum J, Moiseyenko V, Jones SE, Miles D, Bell D, Rosso R, Mauriac L, Osterwalder B, Burger H-U, Laws S (2001) Randomized, open-label, phase II trial of oral capecitabine (Xeloda) vs. a reference arm of intravenous CMF (cyclophosphamide, methotrexate and 5-fluorouracil) as first-line therapy for advanced/metastatic breast cancer. Annals of Oncology 12:1247-1254

9. Paridaens RJ, Dirix LY, Beex LV, Nooij M, Cameron DA, Cufer T, Piccart MJ, Bogaerts J, Therasse P (2008) Phase III study comparing exemestane with tamoxifen as first-line hormonal treatment of metastatic breast cancer in postmenopausal women: the European Organisation for Research and Treatment of Cancer Breast Cancer Cooperative Group. J Clin Oncol 26 (30):4883-4890. doi:10.1200/JCO.2007.14.4659

10. Robert NJ, Saleh MN, Paul D, Generali D, Gressot L, Copur MS, Brufsky AM, Minton SE, Giguere JK, Smith JW, 2nd, Richards PD, Gernhardt D, Huang X, Liau KF, Kern KA, Davis J (2011) Sunitinib plus paclitaxel versus bevacizumab plus paclitaxel for first-line treatment of patients with advanced breast cancer: a phase III, randomized, open-label trial. Clin Breast Cancer 11 (2):82-92. doi:10.1016/j.clbc.2011.03.005

11. Robert NJ, Dieras V, Glaspy J, Brufsky AM, Bondarenko I, Lipatov ON, Perez EA, Yardley DA, Chan SY, Zhou X, Phan SC, O'Shaughnessy J (2011) RIBBON-1: randomized, double-blind, placebo-controlled, phase III trial of chemotherapy with or without bevacizumab for first-line treatment of human epidermal growth factor receptor 2-negative, locally recurrent or metastatic breast cancer. J Clin Oncol 29 (10):1252-1260. doi:10.1200/JCO.2010.28.0982
12. Robertson JF, Lindemann JP, Llombart-Cussac A, Rolski J, Feltl D, Dewar J, Emerson L, Dean A, Ellis MJ (2012) Fulvestrant 500 mg versus anastrozole 1 mg for the first-line treatment of advanced breast cancer: follow-up analysis from the randomized 'FIRST' study. Breast cancer research and treatment 136 (2):503-511. doi:10.1007/s10549-012-2192-4
13. Vici P, Giotta F, Di Lauro L, Sergi D, Vizza E, Mariani L, Latorre A, Pizzuti L, D'Amico C, Giannarelli D, Colucci G (2011) A multicenter phase II randomized trial of docetaxel/gemcitabine versus docetaxel/capecitabine as first-line treatment for advanced breast cancer: a Gruppo Oncologico Italia Meridionale study. Oncology 81 (3-4):230-236. doi:10.1159/000334432
14. Welt A, Marschner N, Lerchenmueller C, Decker T, Steffens CC, Koehler A, Depenbusch R, Busies S, Hegewisch-Becker S (2016) Capecitabine and bevacizumab with or without vinorelbine in first-line treatment of HER2/neu-negative metastatic or locally advanced breast cancer: final efficacy and safety data of the randomised, open-label superiority phase 3 CARIN trial. Breast cancer research and treatment 156 (1):97-107. doi:10.1007/s10549-016-3727-x
15. Yardley DA, Burris HA, 3rd, Spigel DR, Clark BL, Vazquez E, Shipley D, Barton J, Thompson D, Montes I, Greco FA, Hainsworth JD (2009) A phase II randomized crossover study of liposomal doxorubicin versus weekly docetaxel in the first-line treatment of women with metastatic breast cancer. Clin Breast Cancer 9 (4):247-252. doi:10.3816/CBC.2009.n.042
16. Yardley DA, Bosserman LD, O'Shaughnessy JA, Harwin WN, Morgan SK, Priego VM, Peacock NW, Bass JD, Burris HA, 3rd, Hainsworth JD (2015) Paclitaxel, bevacizumab, and everolimus/placebo as first-line treatment for patients with metastatic HER2-negative breast cancer: a randomized placebo-controlled phase II trial of the Sarah Cannon Research Institute. Breast cancer research and treatment 154 (1):89-97. doi:10.1007/s10549-015-3599-5
17. Bachelot T, Bajard A, Ray-Coquard I, Provencal J, Coeffic D, Agostini C, Boisseau M, Kaphan R, Dramais D, Oprea C, Ferri-Dessens RM, Guastalla JP, Perol D (2011) Final results of ERASME-4: a randomized trial of first-line docetaxel plus either capecitabine or epirubicin for metastatic breast cancer. Oncology 80 (3-4):262-268. doi:10.1159/000329066
18. Dixon AR, Jackson L, Chan S, Haybittle J, Blamey RW (1992) A randomised trial of second-line hormone vs single agent chemotherapy in tamoxifen resistant advanced breast cancer. Br J Cancer 66 (2):402-404
19. Fountzilas G, Dafni U, Dimopoulos MA, Koutras A, Skarlos D, Papakostas P, Gogas H, Bafaloukos D, Kalogera-Fountzila A, Samantas E, Briasoulis E, Pectasides D, Maniadakis N, Matsiakou F, Aravantinos G, Papadimitriou C, Karina M, Christodoulou C, Kosmidis P, Kalofonos HP (2009) A randomized phase III study comparing three anthracycline-free taxane-based regimens, as first line chemotherapy, in metastatic breast cancer: a Hellenic Cooperative Oncology Group study. Breast cancer research and treatment 115 (1):87-99. doi:10.1007/s10549-008-0047-9
20. Ghosn M, Aftimos P, Farhat FS, Kattan JG, Hanna C, Haddad N, Nasr F, Chahine G (2011) A phase II randomized study comparing navelbine and capecitabine (Navcap) followed either by Navcap or by weekly docetaxel in the first-line treatment of HER-2/neu negative metastatic breast cancer. Med Oncol 28 Suppl 1:S142-151. doi:10.1007/s12032-010-9754-2
21. Heidemann E, Stoeger H, Souchon R, Hirschmann WD, Bodenstein H, Oberhoff C, Fischer JT, Schulze M, Clemens M, Andreesen R, Mahlke M, Konig M, Scharl A, Fehnle K, Kaufmann M (2002) Is first-line single-agent mitoxantrone in the treatment of high-risk metastatic breast cancer patients as effective as combination chemotherapy? No difference in survival but higher quality of life were found in a multicenter randomized trial. Ann Oncol 13 (11):1717-1729

22. Kaufmann M, Bajetta E, Dirix LY, Fein LE, Jones SE, Zilembo N, Dugardyn JL, Nasurdi C, Mennel RG, Cervek J, Fowst C, Polli A, di Salle E, Arkhipov A, Piscitelli G, Miller LL, Massimini G (2000) Exemestane is superior to megestrol acetate after tamoxifen failure in postmenopausal women with advanced breast cancer: results of a phase III randomized double-blind trial. The Exemestane Study Group. J Clin Oncol 18 (7):1399-1411
23. Miller K, Wang M, Gralow J, Dickler M, Cobleigh M, Perez EA, Shenkier T, Cella D, Davidson NE (2007) Paclitaxel plus bevacizumab versus paclitaxel alone for metastatic breast cancer. The New England journal of medicine 357 (26):2666-2676. doi:10.1056/NEJMoa072113
24. Ackland SP, Anton A, Breitbach GP, Colajori E, Tursi JM, Delfino C, Efremidis A, Ezzat A, Fittipaldo A, Kolaric K, Lopez M, Viaro D, group Hs (2001) Dose-intensive epirubicin-based chemotherapy is superior to an intensive intravenous cyclophosphamide, methotrexate, and fluorouracil regimen in metastatic breast cancer: a randomized multinational study. J Clin Oncol 19 (4):943-953
25. Baselga J, Segalla JG, Roche H, Del Giglio A, Pinczowski H, Ciruelos EM, Filho SC, Gomez P, Van Eyll B, Bermejo B, Llombart A, Garicochea B, Duran MA, Hoff PM, Espie M, de Moraes AA, Ribeiro RA, Mathias C, Gil Gil M, Ojeda B, Morales J, Kwon Ro S, Li S, Costa F (2012) Sorafenib in combination with capecitabine: an oral regimen for patients with HER2-negative locally advanced or metastatic breast cancer. J Clin Oncol 30 (13):1484-1491. doi:10.1200/JCO.2011.36.7771
26. Bergh J, Bondarenko IM, Lichinitser MR, Liljegren A, Greil R, Voytko NL, Makhson AN, Cortes J, Lortholary A, Bischoff J, Chan A, Delaloge S, Huang X, Kern KA, Giorgetti C (2012) First-line treatment of advanced breast cancer with sunitinib in combination with docetaxel versus docetaxel alone: results of a prospective, randomized phase III study. J Clin Oncol 30 (9):921-929. doi:10.1200/JCO.2011.35.7376
27. Brufsky A, Hoelzer K, Beck T, Whorf R, Keaton M, Nadella P, Krill-Jackson E, Kroener J, Middleman E, Frontiera M, Paul D, Panella T, Bromund J, Zhao L, Orlando M, Tai F, Marciniak MD, Obasaju C, Hainsworth J (2011) A randomized phase II study of paclitaxel and bevacizumab with and without gemcitabine as first-line treatment for metastatic breast cancer. Clin Breast Cancer 11 (4):211-220. doi:10.1016/j.clbc.2011.03.019
28. Buzdar AU, Jones SE, Vogel CL, Wolter J, Plourde P, Webster A (1997) A phase III trial comparing anastrozole (1 and 10 milligrams), a potent and selective aromatase inhibitor, with megestrol acetate in postmenopausal women with advanced breast carcinoma. Arimidex Study Group. Cancer 79 (4):730-739
29. Buzdar A, Douma J, Davidson N, Elledge R, Morgan M, Smith R, Porter L, Nabholtz J, Xiang X, Brady C (2001) Phase III, multicenter, double-blind, randomized study of letrozole, an aromatase inhibitor, for advanced breast cancer versus megestrol acetate. J Clin Oncol 19 (14):3357-3366
30. Campone M, Dobrovolskaya N, Tjulandin S, Chen SC, Fourie S, Mefti F, Konstantinova M, Lefresne F, Meheust N, Jassem J (2013) A three-arm randomized phase II study of oral vinorelbine plus capecitabine versus oral vinorelbine and capecitabine in sequence versus docetaxel plus capecitabine in patients with metastatic breast cancer previously treated with anthracyclines. Breast J 19 (3):240-249. doi:10.1111/tbj.12098
31. Chan S, Romieu G, Huober J, Delozier T, Tubiana-Hulin M, Schneeweiss A, Lluch A, Llombart A, du Bois A, Kreienberg R, Mayordomo JI, Anton A, Harrison M, Jones A, Carrasco E, Vaury AT, Frimodt-Moller B, Fumoleau P (2009) Phase III study of gemcitabine plus docetaxel compared with capecitabine plus docetaxel for anthracycline-pretreated patients with metastatic breast cancer. J Clin Oncol 27 (11):1753-1760. doi:10.1200/JCO.2007.15.8485
32. Chia S, Gradishar W, Mauriac L, Bines J, Amant F, Federico M, Fein L, Romieu G, Buzdar A, Robertson JF, Brufsky A, Possinger K, Rennie P, Sapunar F, Lowe E, Piccart M (2008) Double-blind, randomized placebo controlled trial of fulvestrant compared with exemestane after prior nonsteroidal aromatase inhibitor therapy in postmenopausal women with hormone receptor-positive, advanced breast cancer: results from EFECT. J Clin Oncol 26 (10):1664-1670. doi:10.1200/JCO.2007.13.5822

33. Cristofanilli M, Turner NC, Bondarenko I, Ro J, Im SA, Masuda N, Colleoni M, DeMichele A, Loi S, Verma S, Iwata H, Harbeck N, Zhang K, Theall KP, Jiang Y, Bartlett CH, Koehler M, Slamon D (2016) Fulvestrant plus palbociclib versus fulvestrant plus placebo for treatment of hormone-receptor-positive, HER2-negative metastatic breast cancer that progressed on previous endocrine therapy (PALOMA-3): final analysis of the multicentre, double-blind, phase 3 randomised controlled trial. Lancet Oncol. doi:10.1016/S1470-2045(15)00613-0
34. Crown JP, Dieras V, Staroslawska E, Yardley DA, Bachelot T, Davidson N, Wildiers H, Fasching PA, Capitain O, Ramos M, Greil R, Cognetti F, Fountzilas G, Blasinska-Morawiec M, Liedtke C, Kreienberg R, Miller WH, Jr., Tassell V, Huang X, Paolini J, Kern KA, Romieu G (2013) Phase III trial of sunitinib in combination with capecitabine versus capecitabine monotherapy for the treatment of patients with pretreated metastatic breast cancer. J Clin Oncol 31 (23):2870-2878. doi:10.1200/JCO.2012.43.3391
35. Del Mastro L, Fabi A, Mansutti M, De Laurentiis M, Durando A, Merlo DF, Bruzzi P, La Torre I, Ceccarelli M, Kazeem G, Marchi P, Boy D, Venturini M, De Placido S, Cognetti F (2013) Randomised phase 3 open-label trial of first-line treatment with gemcitabine in association with docetaxel or paclitaxel in women with metastatic breast cancer: a comparison of different schedules and treatments. BMC cancer 13:164. doi:10.1186/1471-2407-13-164
36. Di Leo A, Jerusalem G, Petruzelka L, Torres R, Bondarenko IN, Khasanov R, Verhoeven D, Pedrini JL, Smirnova I, Lichinitser MR, Pendergrass K, Garnett S, Lindemann JP, Sapunar F, Martin M (2010) Results of the CONFIRM phase III trial comparing fulvestrant 250 mg with fulvestrant 500 mg in postmenopausal women with estrogen receptor-positive advanced breast cancer. J Clin Oncol 28 (30):4594-4600. doi:10.1200/JCO.2010.28.8415
37. Dombernowsky P, Smith I, Falkson G, Leonard R, Panasci L, Bellmunt J, Bezwoda W, Gardin G, Gudgeon A, Morgan M, Fornasiero A, Hoffmann W, Michel J, Hatschek T, Tjabbes T, Chaudri HA, Hornberger U, Trunet PF (1998) Letrozole, a new oral aromatase inhibitor for advanced breast cancer: double-blind randomized trial showing a dose effect and improved efficacy and tolerability compared with megestrol acetate. J Clin Oncol 16 (2):453-461
38. Fountzilas G, Kalofonos HP, Dafni U, Papadimitriou C, Bafaloukos D, Papakostas P, Kalogera-Fountzila A, Gogas H, Aravantinos G, Moulopoulos LA, Economopoulos T, Pectasides D, Maniadakis N, Siafaka V, Briasoulis E, Christodoulou C, Tsavdaridis D, Makrantonakis P, Razis E, Kosmidis P, Skarlos D, Dimopoulos MA (2004) Paclitaxel and epirubicin versus paclitaxel and carboplatin as first-line chemotherapy in patients with advanced breast cancer: a phase III study conducted by the Hellenic Cooperative Oncology Group. Ann Oncol 15 (10):1517-1526. doi:10.1093/annonc/mdh395
39. Gershanovich M, Chaudri HA, Campos D, Lurie H, Bonaventura A, Jeffrey M, Buzzi F, Bodrogi I, Ludwig H, Reichardt P, O'Higgins N, Romieu G, Friederich P, Lassus M (1998) Letrozole, a new oral aromatase inhibitor: randomised trial comparing 2.5 mg daily, 0.5 mg daily and aminoglutethimide in postmenopausal women with advanced breast cancer. Letrozole International Trial Group (AR/BC3). Ann Oncol 9 (6):639-645
40. Hatschek T, Carlsson L, Einbeigi Z, Lidbrink E, Linderholm B, Lindh B, Loman N, Malmberg M, Rotstein S, Soderberg M, Sundquist M, Walz TM, Hellstrom M, Svensson H, Astrom G, Brandberg Y, Carstensen J, Ferno M, Bergh J (2012) Individually tailored treatment with epirubicin and paclitaxel with or without capecitabine as first-line chemotherapy in metastatic breast cancer: a randomized multicenter trial. Breast cancer research and treatment 131 (3):939-947. doi:10.1007/s10549-011-1880-9
41. Howell A, Robertson JF, Quaresma Albano J, Aschermannova A, Mauriac L, Kleeberg UR, Vergote I, Erikstein B, Webster A, Morris C (2002) Fulvestrant, formerly ICI 182,780, is as effective as anastrozole in postmenopausal women with advanced breast cancer progressing after prior endocrine treatment. J Clin Oncol 20 (16):3396-3403
42. Jiang Z, Zhang Q, Shao Z, Shen K, Li L, Feng J, Tong Z, Gu K, Wang X, Xu B, Sun G, Chen H, Rukazenkov Y (2014) A phase III study of fulvestrant 500 mg versus 250 mg in postmenopausal Chinese women with

advanced breast cancer and disease progression following failure on prior antiestrogen or aromatase inhibitor therapy: Supporting superior clinical benefit for the 500 mg dose. Proceedings of the Thirty-Seventh Annual CTRC-AACR San Antonio Breast Cancer Symposium
43. Jonat W, Howell A, Blomqvist C, Eiermann W, Winblad G, Tyrrell C, Mauriac L, Roche H, Lundgren S, Hellmund R, Azab M (1996) A randomised trial comparing two doses of the new selective aromatase inhibitor anastrozole (Arimidex) with megestrol acetate in postmenopausal patients with advanced breast cancer. Eur J Cancer 32A (3):404-412
44. Jones SE, Erban J, Overmoyer B, Budd GT, Hutchins L, Lower E, Laufman L, Sundaram S, Urba WJ, Pritchard KI, Mennel R, Richards D, Olsen S, Meyers ML, Ravdin PM (2005) Randomized phase III study of docetaxel compared with paclitaxel in metastatic breast cancer. J Clin Oncol 23 (24):5542-5551. doi:10.1200/JCO.2005.02.027
45. Luck HJ, Du Bois A, Loibl S, Schrader I, Huober J, Heilmann V, Beckmann M, Stahler A, Jackisch C, Hubalek M, Richter B, Stickeler E, Eidtmann H, Thomssen C, Untch M, Wollschlager K, Schuster T, von Minckwitz G (2013) Capecitabine plus paclitaxel versus epirubicin plus paclitaxel as first-line treatment for metastatic breast cancer: efficacy and safety results of a randomized, phase III trial by the AGO Breast Cancer Study Group. Breast cancer research and treatment 139 (3):779-787. doi:10.1007/s10549-013-2589-8
46. Martin M, Roche H, Pinter T, Crown J, Kennedy MJ, Provencher L, Priou F, Eiermann W, Adrover E, Lang I, Ramos M, Latreille J, Jagiello-Gruszfeld A, Pienkowski T, Alba E, Snyder R, Almel S, Rolski J, Munoz M, Moroose R, Hurvitz S, Banos A, Adewoye H, Hei YJ, Lindsay MA, Rupin M, Cabaribere D, Lemmerick Y, Mackey JR, investigators T (2011) Motesanib, or open-label bevacizumab, in combination with paclitaxel, as first-line treatment for HER2-negative locally recurrent or metastatic breast cancer: a phase 2, randomised, double-blind, placebo-controlled study. Lancet Oncol 12 (4):369-376. doi:10.1016/S1470-2045(11)70037-7
47. Martin M, Martinez N, Ramos M, Calvo L, Lluch A, Zamora P, Munoz M, Carrasco E, Caballero R, Garcia-Saenz JA, Guerra E, Caronia D, Casado A, Ruiz-Borrego M, Hernando B, Chacon JI, De la Torre-Montero JC, Jimeno MA, Heras L, Alonso R, De la Haba J, Pita G, Constenla M, Gonzalez-Neira A (2015) Standard versus continuous administration of capecitabine in metastatic breast cancer (GEICAM/2009-05): a randomized, noninferiority phase II trial with a pharmacogenetic analysis. Oncologist 20 (2):111-112. doi:10.1634/theoncologist.2014-0379
48. Ohno S, Rai Y, Iwata H, Yamamoto N, Yoshida M, Iwase H, Masuda N, Nakamura S, Taniguchi H, Kamigaki S, Noguchi S (2010) Three dose regimens of fulvestrant in postmenopausal Japanese women with advanced breast cancer: results from a double-blind, phase II comparative study (FINDER1). Ann Oncol 21 (12):2342-2347. doi:10.1093/annonc/mdq249
49. Osborne CK, Pippen J, Jones SE, Parker LM, Ellis M, Come S, Gertler SZ, May JT, Burton G, Dimery I, Webster A, Morris C, Elledge R, Buzdar A (2002) Double-blind, randomized trial comparing the efficacy and tolerability of fulvestrant versus anastrozole in postmenopausal women with advanced breast cancer progressing on prior endocrine therapy: results of a North American trial. J Clin Oncol 20 (16):3386-3395
50. Papadimitriou CA, Kalofonos H, Zagouri F, Papakostas P, Bozas G, Makatsoris T, Dimopoulos MA, Fountzilas G (2009) Weekly docetaxel with or without gemcitabine as second-line chemotherapy in paclitaxel-pretreated patients with metastatic breast cancer: a randomized phase II study conducted by the Hellenic Co-Operative Oncology Group. Oncology 77 (3-4):212-216. doi:10.1159/000236021
51. Pritchard KI, Rolski J, Papai Z, Mauriac L, Cardoso F, Chang J, Panasci L, Ianuli C, Kahan Z, Fukase K, Lindemann JP, Macpherson MP, Neven P (2010) Results of a phase II study comparing three dosing regimens of fulvestrant in postmenopausal women with advanced breast cancer (FINDER2). Breast cancer research and treatment 123 (2):453-461. doi:10.1007/s10549-010-1022-9
52. Rugo HS, Campone M, Amadori D, Aldrighetti D, Conte P, Wardley A, Villanueva C, Melisko M, McHenry MB, Liu D, Lee F, Pivot X (2013) A randomized, phase II, three-arm study of two schedules of ixabepilone

or paclitaxel plus bevacizumab as first-line therapy for metastatic breast cancer. Breast cancer research and treatment 139 (2):411-419. doi:10.1007/s10549-013-2552-8
53. Rugo HS, Barry WT, Moreno-Aspitia A, Lyss AP, Cirrincione C, Leung E, Mayer EL, Naughton M, Toppmeyer D, Carey LA, Perez EA, Hudis C, Winer EP (2015) Randomized Phase III Trial of Paclitaxel Once Per Week Compared With Nanoparticle Albumin-Bound Nab-Paclitaxel Once Per Week or Ixabepilone With Bevacizumab As First-Line Chemotherapy for Locally Recurrent or Metastatic Breast Cancer: CALGB 40502/NCCTG N063H (Alliance). J Clin Oncol 33 (21):2361-2369. doi:10.1200/JCO.2014.59.5298
54. Smorenburg CH, de Groot SM, van Leeuwen-Stok AE, Hamaker ME, Wymenga AN, de Graaf H, de Jongh FE, Braun JJ, Los M, Maartense E, van Tinteren H, Nortier JW, Seynaeve C (2014) A randomized phase III study comparing pegylated liposomal doxorubicin with capecitabine as first-line chemotherapy in elderly patients with metastatic breast cancer: results of the OMEGA study of the Dutch Breast Cancer Research Group BOOG. Ann Oncol 25 (3):599-605. doi:10.1093/annonc/mdt588
55. Stockler MR, Harvey VJ, Francis PA, Byrne MJ, Ackland SP, Fitzharris B, Van Hazel G, Wilcken NR, Grimison PS, Nowak AK, Gainford MC, Fong A, Paksec L, Sourjina T, Zannino D, Gebski V, Simes RJ, Forbes JF, Coates AS (2011) Capecitabine versus classical cyclophosphamide, methotrexate, and fluorouracil as first-line chemotherapy for advanced breast cancer. J Clin Oncol 29 (34):4498-4504. doi:10.1200/JCO.2010.33.9101
56. Wang J, Xu B, Yuan P, Ma F, Li Q, Zhang P, Cai R, Fan Y, Luo Y, Li Q (2015) Capecitabine combined with docetaxel versus vinorelbine followed by capecitabine maintenance medication for first-line treatment of patients with advanced breast cancer: Phase 3 randomized trial. Cancer 121 (19):3412-3421. doi:10.1002/cncr.29492
57. Xu B, Jiang Z, Shao Z, Wang J, Feng J, Song S, Chen Z, Gu K, Yu S, Zhang Y, Wang C, Zhang F, Yang J (2011) Fulvestrant 250 mg versus anastrozole for Chinese patients with advanced breast cancer: results of a multicentre, double-blind, randomised phase III trial. Cancer Chemother Pharmacol 67 (1):223-230. doi:10.1007/s00280-010-1483-x
58. Yardley DA, Noguchi S, Pritchard KI, Burris HA, 3rd, Baselga J, Gnant M, Hortobagyi GN, Campone M, Pistilli B, Piccart M, Melichar B, Petrakova K, Arena FP, Erdkamp F, Harb WA, Feng W, Cahana A, Taran T, Lebwohl D, Rugo HS (2013) Everolimus plus exemestane in postmenopausal patients with HR(+) breast cancer: BOLERO-2 final progression-free survival analysis. Adv Ther 30 (10):870-884. doi:10.1007/s12325-013-0060-1
59. Paridaens R, Biganzoli L, Bruning P, Klijn JG, Gamucci T, Houston S, Coleman R, Schachter J, Van Vreckem A, Sylvester R, Awada A, Wildiers J, Piccart M (2000) Paclitaxel versus doxorubicin as first-line single-agent chemotherapy for metastatic breast cancer: a European Organization for Research and Treatment of Cancer Randomized Study with cross-over. J Clin Oncol 18 (4):724-733
60. NICE (2012) Process and methods guides. The guidelines manual. National Institute for Health and Care Excellence
